# Effects of Patient Empowerment Programme (PEP) on Clinical Outcomes and Health Service Utilization in Type 2 Diabetes Mellitus in Primary Care: An Observational Matched Cohort Study

**DOI:** 10.1371/journal.pone.0095328

**Published:** 2014-05-01

**Authors:** Carlos K. H. Wong, William C. W. Wong, Cindy L. K. Lam, Y. F. Wan, Winnie H. T. Wong, K. L. Chung, Daisy Dai, Eva L. H. Tsui, Daniel Y. T. Fong

**Affiliations:** 1 Department of Family Medicine and Primary Care, The University of Hong Kong, Hong Kong; 2 Integrated Care Programs, Hospital Authority Head Office, Hong Kong Hospital Authority, Kowloon, Hong Kong; 3 Primary and Community Services, Hospital Authority Head Office, Hong Kong Hospital Authority, Kowloon, Hong Kong; 4 Statistics and Workforce Planning, Hospital Authority Head Office, Hong Kong Hospital Authority, Kowloon, Hong Kong; 5 School of Nursing, The University of Hong Kong, Hong Kong; Endocrine Research Center (Firouzgar), Institute of Endocrinology and Metabolism, Iran (Republic of Islamic)

## Abstract

**Background:**

To evaluate the effects of a large population-based patient empowerment programme (PEP) on clinical outcomes and health service utilization rates in type 2 diabetes mellitus (T2DM) patients in the primary care setting.

**Research Design and Subjects:**

A stratified random sample of 1,141 patients with T2DM enrolled to PEP between March and September 2010 were selected from general outpatient clinics (GOPC) across Hong Kong and compared with an equal number of T2DM patients who had not participated in the PEP (non-PEP group) matched by age, sex and HbA_1C_ level group.

**Measures:**

Clinical outcomes of HbA_1c_, SBP, DBP and LDL-C levels, and health service utilization rates including numbers of visits to GOPC, specialist outpatient clinics (SOPC), emergency department (ED) and inpatient admissions, were measured at baseline and at 12-month post-recruitment. The effects of PEP on clinical outcomes and health service utilization rates were assessed by the difference-in-difference estimation, using the generalized estimating equation models.

**Results:**

Compared with non-PEP group, PEP group achieved additional improvements in clinical outcomes over the 12-month period. A significantly greater percentage of patients in the PEP group attained HbA_1C_≤7% or LDL-C≤2.6 mmol/L at 12-month follow-up compared with the non-PEP group. PEP group had a mean 0.813 fewer GOPC visits in comparison with the non-PEP group.

**Conclusions:**

PEP was effective in improving the clinical outcomes and reduced the general outpatient clinic utilization rate over a 12-month period. Empowering T2DM patients on self-management of their disease can enhance the quality of diabetes care in primary care.

**Trial Registration:**

ClinicalTrials.gov NCT01935349

## Introduction

Diabetes mellitus is a chronic disease that requires long-term lifestyle modifications and medical care. Improved metabolic control through diet and physical exercise, with anti-diabetes medications, could effectively reduce the risk of complications [1]. However, patients often failed to effectively manage their conditions [2], [3], and merely 16.2% reported they followed self-management recommendations completely [4]. This highlights the need for an effective approach to engaging patients in self-management practices necessary for optimal disease control.

‘Patient empowerment’ refers to “a process where people gain greater control over decisions affecting their health” [5]. This concept has been proposed for managing diabetes. The principle is to enable patients to be the primary decision maker in managing their health condition, based on the notions that patients are more motivated to initiate and sustain behavioral changes of their choice than changes prescribed by others [6], [7].This approach requires a collaborative relationship between the patient and the healthcare provider, where latter would serve to facilitate the patient in making informed decisions by providing necessary resources. Currently, there is no clear consensus on the optimal composition and organization of patient empowerment programme (PEP) for optimal outcomes. Common topics included in the curriculum are diet, physical exercise, self-monitoring (blood or urine glucose), treatment adherence, foot care, and management of complications and treatment side effects; while behavioral change techniques including emotional coping, problem-solving, goal setting and action planning are frequently used [7]–[10].

Systemic reviews of randomized trials showed that self-management education with comprehensive lifestyle interventions improved glycemic and cardiovascular risk factor control, yet high-quality long-term studies were lacking [11]–[13]. Recently, two large-scale multicenter randomized controlled trials were completed. The DESMOND (Diabetes Education and Self Management for Ongoing and Newly Diagnosed) trial was a group-based structured education program involving 824 patients with newly diagnosed diabetes in the UK. Although the trial reported positive impacts of education on weight, physical activity, smoking status, and depression scores at one year, there were not maintained at three years and no significant improvements in glycemic control at either follow-up [14]–[16]. The one-off education program was considered insufficient to promote enduring lifestyle changes in patients. The ROMEO (Rethink Organisation to iMprove Education and Outcomes) study, conducted in a sample of 815 type 2 diabetes patients from the secondary care setting, reported that ongoing self-management support provided highly favorable and sustained effects on metabolic control, along with improvements in knowledge, health behaviors, and quality of life in patients with established diabetes [17].

Given the substantial disease burden diabetes imposes on individuals and society, it would be of interest to study the impact of PEPs on health service utilization. Several published studies demonstrated that diabetes self-management training programmes led to fewer hospitalizations, and decreased overall healthcare utilization and costs [18], [19]. Although results from the few Asian studies available supported the transferability of structured education programmes for use in non-Western populations [20]–[22], the current evidence underlines the need for additional well-designed, long-term and culturally-adapted studies. Comprehensive evaluation of these programmes’ effects in the real-life setting would be a valuable addition to the existing literature, which is largely based on clinical trials conducted in academic or medical centers.

The aims of this observational matched cohort study were to evaluate the effectiveness of the PEP at patients’ individual level, and to provide the pre- and 12-month–post programme differences in the outcomes of metabolic control and health service utilization, and then compared the outcome differences between patients with and without undertaking the PEP in a primary care setting. The study provided much needed translational evidence of diabetes self-management education in the real-world setting.

## Methods

The protocol for this trial and supporting TREND checklist are available as supporting information; see Protocol S1 and Checklist S1.

### Setting of Patient Empowerment Programme (PEP)

The Hong Kong Hospital Authority that is responsible for all the public medical services in Hong Kong, has launched the large-population based PEP in 2010 for patients with diabetes as a way to enhance the quality of chronic disease management in primary care in Hong Kong. Specifically, the key objectives of the PEP were: 1) to provide patients with a combination of knowledge and skills and to increase their awareness regarding their own disease conditions so that they can make conscious decisions and act in their own self-interest; 2) to facilitate autonomous self-regulation so that the patients’ potential for health and wellness can be maximized; and, 3) to promote private-public partnership for the service delivery models in patients with chronic diseases.

Two non-government organizations (NGOs) highly experienced in providing community medical services and health education were invited to participate in this programme and deliver the training sessions in the first year. This programme was intended for patients receiving ambulatory care for type 2 diabetes mellitus (T2DM) with diet and/or oral treatment regimen at general outpatient clinics or family medicine specialist clinics of the hospital authority. Patients with the advanced diabetic complications such as severe heart failure, end stage renal failure, and advanced eye diseases were excluded. By following such criteria, clinicians at general outpatient clinics or family medicine specialist clinics referred eligible patients to join PEP.

The study aims and objectives were explained in the information letter to patients, and written informed consent was obtained from the PEP participants. The PEP participants also completed the registration of the electronic platform which documented the participant profile, the participants’ attendance under the PEP, pre- and post-programme assessments, follow-up activities, end-of-programme summary and information exchange for continuation of care. Upon the completion of all registration procedures (i.e. given of informed consent, registration of electronic platform, and completion of pre-programme assessment), a patient is considered to be successfully enrolled into PEP. The curriculum of the PEP included both generic self-efficacy enhancement and lifestyle modification component, as well as disease-specific knowledge and skills component. Generic sessions covered the importance of self-management and behavior modification, healthy diet and regular exercise habit, goal setting and problem solving skills, sharing on self-monitoring experience, stress coping management, psychosocial support and networking, and communications with healthcare professionals. Disease-specific sessions, with a total duration of 300 minutes, covered comprehensive information about diabetes, responsibility of self-care management, medications in diabetes control, and contingency management on hypo- and hyperglycemia. Each PEP session was facilitated by one health care professional with recognized specialty training in diabetes management and education.

### Subjects

All subjects with T2DM who had attended at least one PEP session and had post-assessment conducted at 12 months from baseline were included in the outcome evaluation. The T2DM subjects were identified with the International Classification of Primary Care-2 (ICPC-2) code of ‘T90’, through the clinical management system database of Hong Kong Hospital Authority. A total of 2,407 T2DM subjects, who had enrolled into PEP and attended at least one PEP session between 1 March 2010 and 30 September 2010, were included in the evaluation of the clinical outcomes of care and service utilization rates. 1,141 subjects stratified by age (<60, 60–70 and >70 years of age), sex and disease severity (glycated haemoglobin A_1c_ [HbA_1c_] ≤7%, HbA_1c_ 7.1%–8.4% and HbA_1c_>8.4%) were randomly selected for this evaluation of PEP effectiveness study. Non-PEP participants who had been followed-up in the Hong Kong Hospital Authority general outpatient clinics (GOPC) or family medicine specialist clinics for more than 12 months before 30 September 2010 but had not taken part in PEP were defined as non-PEP controls. To off-set the cohort or placebo effect of the intervened group, 1,141 T2DM adults were matched to PEP subjects on age, sex and HbA1c groups as the control group of the study. We defined the subjects as having hypertension and diabetic complications according to the diagnosis coding system of The International Classification of Diseases, Ninth Revision, Clinical Modification (ICD-9-CM) and ICPC-2.

Ethics approval of this study was granted by the Institutional Review Board of the University of Hong Kong/Hospital Authority Hong Kong West Cluster and clinical trial registry (Clinical trial number and registry: NCT01935349, ClinicalTrials.gov). Participants provided their written informed consent to participate in this study. The ethics committees approved this consent procedure and this study before enrolment of participants started.

### Outcome Measures

This study examined two broad categories of outcomes: quality of care and health service utilization. Quality of clinical outcome for diabetes management including HbA_1c_, blood pressure and LDL-C were evaluated. We hypothesized that the implementation of PEP would significantly reduce HbA_1c_ (unit in %), blood pressure (unit in mmHg) and LDL-C (unit in mmol/L) compared with usual care in non-PEP subjects. Binary outcomes were constructed to indicate the proportion of patients achieving treatment targets set out in the American Diabetes Association’s Standards of Medical Care in Diabetes (i.e. HbA_1c_≤7%, systolic blood pressure [SBP] ≤130 mmHg, diastolic blood pressure [DBP] ≤80 mmHg, blood pressure ≤130/80 mmHg, LDL-C ≤2.6 mmol/L) [10]. Health service utilization pattern was quantified by four categories of doctor visits: general outpatient clinic (GOPC) visits, specialist outpatient clinic (SOPC) visits, emergency department (ED) visits, and inpatient admissions in the 12 months before and 12 months after enrolment to the PEP at the patient-level.

### Data Analysis

Descriptive statistics were used to calculate the mean and standard deviation of clinical outcomes, the proportion of subjects achieving the target goals, and the service utilization rates (mean number of GOPC visits, SOPC visits, ED visits and inpatient admissions) at baseline and 12 months after PEP enrolment for participant group, and at baseline and 12 months after baseline assessment for non-PEP participant group.

We evaluated the within-subject changes after programme intervention, and then determined any difference between PEP participants and non-PEP participants. Within-subject changes in clinical outcomes (HbA_1c_, blood pressure and LDL-C) and service utilization rates (mean number of GOPC visits, SOPC visits, ED visits and inpatient admissions) from baseline to 12 months post-recruitment were analysed by paired t-test for continuous outcomes. The differences in target achievement rates between pre- and post-programme recruitment results were tested by McNemar test for binary outcomes. Unadjusted difference-in-difference estimates of the changes in clinical outcomes and service utilization were reported, and for each of these measures, their differences between the PEP and non-PEP groups were analysed by t-test for continuous outcomes, and Chi square tests for difference in proportions.

To assess the effects of PEP on clinical outcomes of care over time while accounting for within-subject correlation with repeated measurements, we constructed separate generalized estimating equation (GEE) models assigning clinical outcomes (HbA_1c_, SBP, DBP, LDL-C) as dependent variables with an identity link function, assigning targeted outcomes (HbA_1c_≤7%, SBP≤130 mmHg, DBP≤80 mmHg, LDL-C≤2.6 mmol/L) as dependent variables with a binary logistic link function, and assigning health service utilization rates as dependent variables with Poisson loglinear link function. Owing to age, sex and HbA_1c_ level matching, differences in socio-demographic and clinical characteristics at baseline between the PEP and non-PEP subjects were mostly insignificant or small (Table 1), thus these characteristics were not controlled for in the adjusted analyses of difference-in-difference in the following GEE models:

**Table 1:** Baseline Characteristics of Participants in PEP group and non-participants in non-PEP group.

	PEP (N = 1,141)	Non-PEP (N = 1,141)	P-value
Mean Age± SD	64.25±10.01	64.93±11.41	0.129
Sex (n, %)			1.000
Male	567 (50%)	567 (50%)	
Female	574 (50%)	574 (50%)	
Smoking status			0.591
Non-smoker	851 (75.4%)	575 (74.3%)	
Ever smoking	278 (24.6%)	199 (25.7%)	
Alcohol status			0.149
Non-drinker	869 (77.1%)	552 (74.2%)	
Ever drinking	258 (22.9%)	192 (25.8%)	
Clinical Characteristics			
Duration of DM	7.08±6.07	7.58±6.24	0.057
Presence of diabetic complication	94 (8.2%)	124 (10.9%)	0.033*
Hypertension	927 (81.2%)	867 (76.0%)	0.002*
Treatment Modality			0.207
Diet only	137 (12.0%)	118 (10.3%)	
Oral and/or insulin treated	1004 (88.0%)	1023 (89.7%)	

PEP = Patient Empowerment Programme; SD = Standard Deviation; DM = Diabetes Mellitus.

*Statistically different (P<0.05) by independent t-test or Chi-square test.



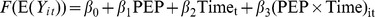
where *Y_it_* is the outcome of interest in *i* participant and *t* time period (Time = 1 equating 12-month follow-up, Time = 0 equating baseline), PEP is a dummy variable (PEP = 1 equating PEP group, PEP = 0 equating non-PEP group) and *F* is a link function for the GEE model. Adjusted difference-in-difference estimates of the impacts of PEP implementation on continuous clinical outcomes were the coefficient on the interaction of time and PEP, defined as *β_3_*. Adjusted difference-in-difference estimates of the effect of intervention on binary and count data outcomes are described in details previously [23]. An exchangeable correlation structure for the within-cluster correlation matrix was assumed in GEE models.

All statistical analyses were performed using STATA Version 12.0 (StataCorp LP. College Station, Tex). All significance tests were two-tailed and findings with a p-value less than 0.05 were considered statistically significant.

## Results

**Figure 1:**
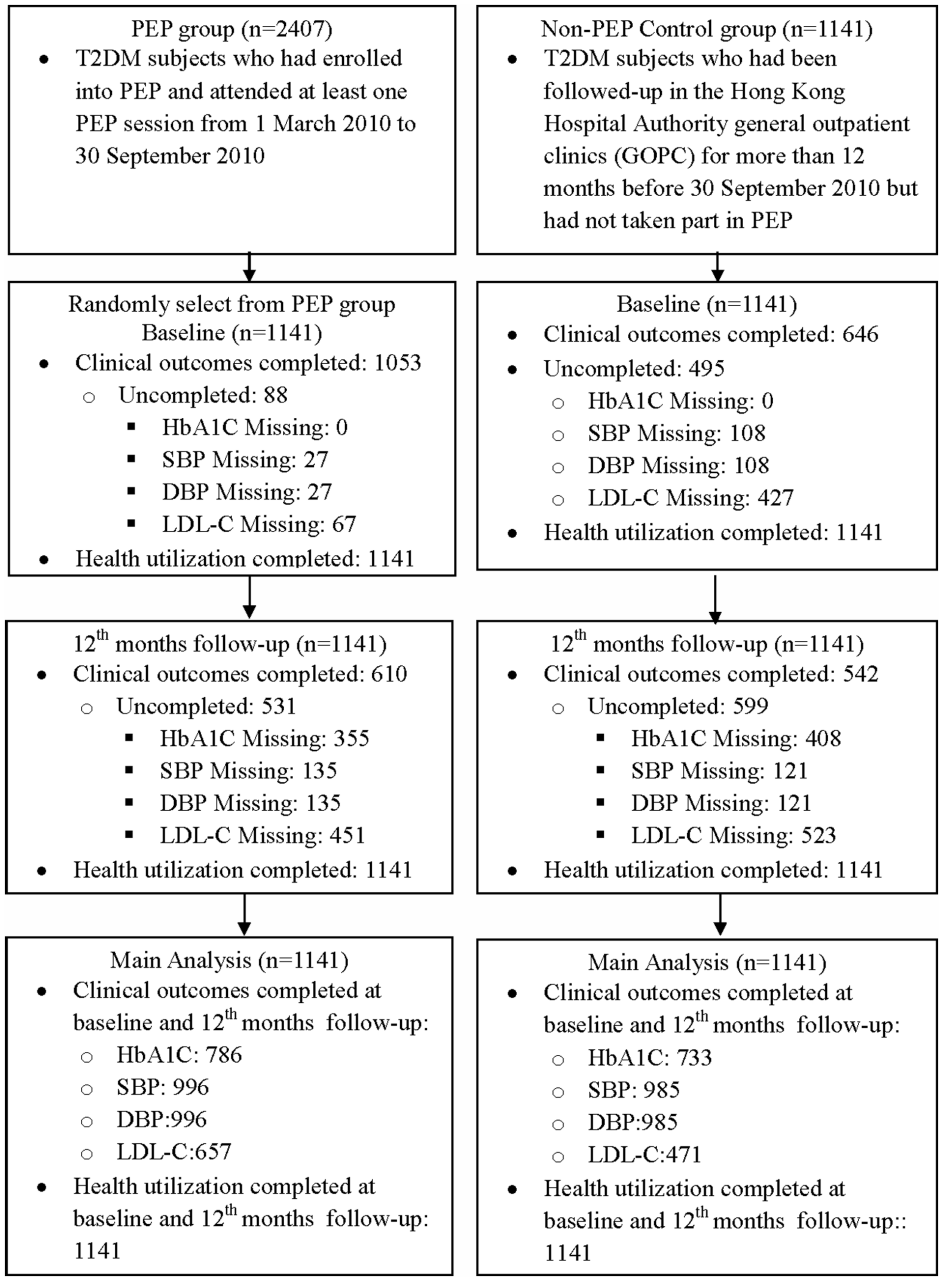
Flow of Participants on the Subject Assignment, Follow-up and Analysis.

Flow of PEP and non-PEP participants on the subject assignment, follow-up and main analysis is displayed in Figure 1. At baseline, the age and gender distribution of PEP group were similar to those of non-PEP group (Table 1) as expected due to sample matching. More PEP participants had a diagnosis of hypertension than non-PEP participants, but fewer PEP participants had a diagnosis of diabetic complication than non-PEP participants. Slight variations in baseline characteristics for clinical diagnoses between PEP and non-PEP groups were not controlled for in the regression model.

**Figure 2:**
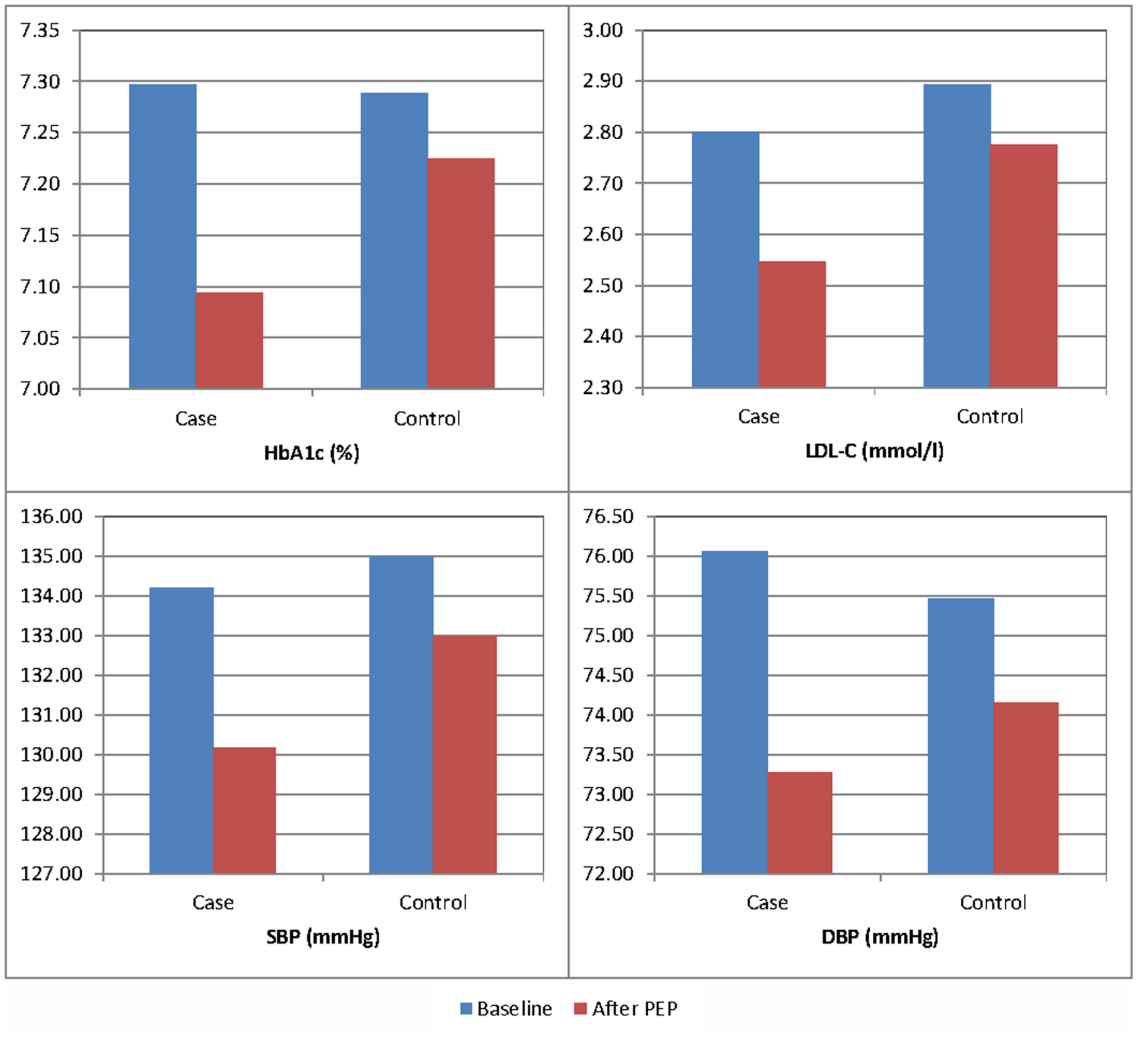
The change from baseline to post-assessment on Clinical Outcomes.

Results of unadjusted analysis in Table 2 and Figure 2 suggest that clinical outcomes in PEP group generally improved significantly after 12 months. The mean HbA_1c_ values decreased significantly over time among PEP group (0.203, P<0.001), in line with the increased proportion of patients with HbA_1c_ ≤7.0% (5.852%, P = 0.001). PEP group had an average decrease of 0.138% in the HbA_1c_ level (95%CI −0.252 to −0.024, P = 0.017) more than non-PEP participants. PEP group achieved a significant decrease in the mean LDL-C value (0.254 mmol/L, P<0.001), and the decrease was significantly more (−0.136 mmol/L, 95%CI −0.223 to −0.048, P<0.001) than that of the non-PEP group. Although there was significant improvement in the proportion of patients reaching the target BP of ≤130/80 in PEP group (SBP: 8.032%, P<0.001; DBP: 8.333%, P<0.001; both SBP and DBP: 7.329%, P<0.001), the change was not significantly greater than those of non-PEP group (SBP: 2.753%, 95%CI 2.094%–3.412%, P = 0.529; DBP: −3.359%, 95%CI 2.693%–4.024%, P = 0.711; SBP/DBP: 1.543%, 95%CI 0.904%–2.181%, P = 0.738). However, this is not the case with mean change in values of SBP and DBP. The reductions in mean SBP and DBP over time among PEP group were significantly greater than those among non-PEP group (SBP: −2.025 mmHg, 95%CI −3.609 to −0.440, P = 0.012; DBP: −1.473 mmHg, 95%CI −2.344 to −0.602, P = 0.012).

**Table 2:** Comparisons of within-subject changes from baseline to post-assessment and Difference-in-difference estimates of the PEP on Clinical Outcomes and 12-month Health Service Utilization.

	PEP				Non-PEP				Unadjusted Difference-in-difference			Adjusted Difference-in-difference		
	Baseline	After PEP	Paired Difference	P-value*	Baseline	After 12 months	Paired Difference	P-value*	Estimate	95%CI	P-value†	Estimate	95%CI	P-value‡
Clinical Outcomes														
HbA1c (Mean ± SD)	7.297±1.245	7.094±0.985	0.203	<0.001	7.289±1.189	7.224±1.141	0.065	0.090	−0.138	(−0.252, −0.024)	0.017*	−0.160	(−0.272, −0.048)	0.005*
≤7% (n, %)	395 (50.254%)	441 (56.107%)	−5.852%	0.003	358 (48.840%)	369 (50.341%)	−1.414%	0.455	4.438%	(4.025,4.851)	<0.001*	5.100%	(0.002,0.100)	0.043*
>7% (n, %)	391 (49.746%)	345 (43.893%)			375 (51.160%)	364 (49.659%)								
SBP (Mean ± SD)	134.209±16.741	130.162±14.611	4.047	<0.001	134.999±17.663	132.977±16.417	2.022	<0.001	−2.025	(−3.609, −0.440)	0.012*	−2.127	(−3.702, −0.553)	0.008*
≤130 mmHg (n, %)	433 (43.474%)	513 (51.506%)	−8.032%	<0.001	399 (40.508%)	451 (45.787%)	−5.279%	0.008	2.753%	(2.094,3.412)	0.018*	3.136%	(−0.021,0.084)	0.240
>130 mmHg (n, %)	563 (56.526%)	483 (48.494%)			586 (59.492%)	534 (54.213%)								
DBP (Mean ± SD)	76.063±9.683	73.274±9.543	2.789	<0.001	75.464±10.904	74.148±10.474	1.316	<0.001	−1.473	(−2.344, −0.602)	<0.001*	−1.512	(−2.382, −0.643)	0.001*
≤80 mmHg (n, %)	684 (68.675%)	767 (77.008%)	−8.333%	<0.001	668 (67.817%)	717 (72.792%)	−4.975%	0.004	3.359%	(2.693,4.024)	0.014*	3.675%	(−0.008,0.081)	0.107
>80 mmHg (n, %)	312 (31.325%)	229 (22.992%)			317 (32.183%)	268 (27.208%)								
SBP/DBP														
≤130/80 mmHg (n, %)	384 (38.554%)	457 (45.884%)	−7.329%	<0.001	350 (35.533%)	407 (41.320%)	−5.787%	0.003	1.543%	(0.904,2.181)	0.195	0.019	(−0.032,0.070)	0.471
>130/80 mmHg (n, %)	612 (61.446%)	539 (54.116%)			635 (64.467%)	578 (58.680%)								
LDL-C (Mean ± SD)	2.801±0.803	2.547±0.702	0.254	<0.001	2.894±0.802	2.776±0.732	0.118	<0.001	−0.136	(−0.223, −0.048)	<0.001*	−0.129	(−0.212, −0.045)	0.002*
≤2.6 mmol/l (n, %)	267 (40.639%)	372 (56.621%)	−15.982%	<0.001	182 (38.641%)	211 (44.798%)	−6.157%	0.011	9.825%	(8.635,11.014)		7.878%	(0.022,0.135)	0.006*
>2.6 mmol/l (n, %)	390 (59.361%)	285 (43.379%)			289 (61.359%)	260 (55.202%)								
12-month Health Service Utilization														
GOPC visits (Mean ± SD)	4.101±2.448	3.794±2.482	0.307	<0.001	4.491±2.413	4.997±2.356	−0.507	<0.001	−0.813	(−0.994, −0.632)	<0.001*	−0.813	(−0.968, −0.659)	<0.001*
Unchanged/Decreased(n, %)	814	71.341%			683	59.860%					<0.001			
Increased (n, %)	327	28.659%			458	40.140%								
SOPC visits (Mean ± SD)	2.052±3.008	2.319±3.529	−0.267	<0.001	2.069±3.523	2.142±3.288	−0.073	0.321	0.195	(−0.015,0.404)	0.069	0.195	(0.103,0.286)	<0.001*
Unchanged/Decreased(n, %)	837	73.357%			847	74.233%					0.634			
Increased (n, %)	304	26.643%			294	25.767%								
ED visits (Mean ± SD)	0.368±0.852	0.358±0.918	0.010	0.749	0.442±0.994	0.427±1.041	0.015	0.678	0.005	(−0.087,0.097)	0.911	0.005	(−0.055,0.066)	0.865
Unchanged/Decreased(n, %)	967	84.750%			953	83.523%					0.422			
Increased (n, %)	174	15.250%			188	16.477%								
Inpatient admissions (Mean ± SD)	0.169±0.550	0.185±0.718	−0.016	0.510	0.218±0.701	0.223±0.712	−0.004	0.857	0.011	(−0.056,0.078)	0.739	0.011	(−0.033,0.056)	0.615
Unchanged/Decreased(n, %)	1044	91.499%			1019	89.308%					0.076			
Increased (n, %)	97	8.501%			122	10.692%								

PEP = Patient Empowerment Programme;HbA1c = Haemoglobin A1c; SBP = Systolic Blood Pressure; DBP = Diastolic Blood Pressure; LDL-C = Low Density Lipoprotein – Cholesterol;

GOPC = General Outpatient Clinic; SOPC = Specialist Outpatient Clinic; ED = Emergency Department; CI = Confidence interval;

*P-value of testing significance using paired t-test.

† P-value of testing significance using independent t-test or Chi-square test, where appropriate.

‡ P-value of testing significance in adjusted difference-in-difference estimate (The result of Generalized Estimating Equation was attached in Table S1).

For service utilization outcomes, there was a statistically significant reduction in the number of GOPC visits in the 12 months after PEP (0.307, P<0.001), which was significantly different from the change found among non-PEP group (−0.813, 95%CI −0.994 to −0.632, P<0.001). The change in the rates of SOPC visits (0.195, 95%CI −0.015–0.404, P = 0.069), ED visits (0.005, 95%CI −0.087–0.097, P = 0.911), and inpatient admissions (0.011, 95%CI −0.056–0.078, P = 0.739) were not significantly different between the PEP and non-PEP groups.

Results of the adjusted GEE analysis with the adjusted difference-in-difference estimates of clinical outcomes and health service utilization are shown in Table 2. After adjusting for within-subject correlation, the PEP group showed a greater reduction in HbA_1c_ (−0.160%, 95%CI −0.272 to −0.048, P = 0.005), SBP (−2.217 mmHg 95%CI −3.702 to −0.553, P = 0.008), DBP (−1.512 mmHg, 95%CI −2.382 to −0.643, P = 0.001) and LDL-C (−0.129 mmol/L, 95%CI −0.212 to −0.045, P = 0.002) levels at 12-month follow-up compared with the non-PEP group. There was a significantly greater increase in the proportion of subjects reaching treatment targets of HbA_1c_ (0.051, 95%CI 0.002–0.100, P = 0.043) and LDL-C (0.079, 95%CI 0.022–0.135, P = 0.006) in the PEP group from baseline to 12-month follow-up, when compared with non-PEP group. For BP outcomes, the increase in the target achievement rate among PEP group was not significantly greater than that among non-PEP group. For health service utilization, PEP was associated with reduced utilization of the GOPC (P<0.001) but paradoxically increased utilization of SOPC (P<0.001) over the 12-month period. Changes in ED visits (0.005, 95%CI −0.055–0.066, P = 0.865) and inpatient admission (0.011, 95%CI −0.033–0.056, P = 0.615) were not statistically significantly different between the PEP and non-PEP groups.

## Discussions

This evaluation study demonstrated in T2DM patients significant associations of PEP participation with improved clinical outcomes in HbA_1c_ and LDL-C and reduced GOPC visits than patients who did not participate in PEP.

### HbA1c Reduction after PEP

Our results found that at 12 months, both the PEP and control groups had made significant improvement in the key diabetes measure, the HbA_1c_ level, suggesting a general improvement in the quality of care for these patients. The PEP group had significantly greater programme 12-month changes than the non-PEP group with a mean excess reduction in HbA_1c_ of 0.16% and a 5.1% more increase in the proportion of patients with HbA_1c_ equal to or less than 7.0%. The magnitude of HbA_1c_ improvement seemed to be smaller than the 0.3% to 1.0% net benefit reported in previous studies comparing structured education programme with usual care [24]–[28]. Our study population was mainly patients with mild-to-moderate diabetes mellitus with nearly 50% of patients having HbA_1c_ less than 7.0%. The modest effect of PEP may be related to the relatively low mean baseline HbA_1c_ levels of our subjects, which allowed for little room for large improvements. A study conducted in Taiwan showed that there were no significant changes in the mean HbA_1c_ associated with self-management education in the overall study population, except a 0.5% drop in those with poorly controlled baseline HbA_1c_ (>7%) who showed after one year [22].

There are published studies of group-based diabetes self-management training that reported improved short-term glycaemic control versus usual care or no formal diabetes education in a primary-care setting. The Expert Patient Education (X-PERT) programme, which was based on theories of empowerment and discovery learning, reported a significant Hb_A1c_ improvement (−0.6% vs 0.1%) at 14 months compared with one-to-one care from a dietician [29]. A self-management programme in Sweden showed a 0.94% and 1.4% improvement in the Hb_Alc_ level versus conventional diabetes care at 1 year and 5 years, respectively [30], [31]. The DESMOND study showed no HbA1c improvements with structured education at one-year or three-year follow up [14], [16]. Compared with the DESMOND study, our study used a similar patient-centered collaborative approach to self-management education but there are some distinct differences. DESMOND was a one-off programme that consisted of six hours of group education [14]. In contrast, the PEP in our study comprised a series of group and individual sessions, each lasting for two hours and for 30 minutes, respectively. Furthermore, participants were followed up by telephone bimonthly for six months upon the completion of PEP sessions. The better outcomes in our study than that of DESMOND could be the result of greater contact time between educators and patients, which has been found to be a strong predictor of improved glycemic control [32], [33].

Besides contact time, the PEP also consists of commonalities of education programmes that have been shown to bring about improved outcomes: 1) coupling diabetes management with behavioral strategies – namely action planning and problem solving [34], [35], and 2) addressing the three key aspects of chronic disease: medical, social and emotional needs of patients [36]. Current guidelines highlight that effective education strategies entail an interactive approach exemplifying the importance of action-oriented goals, coupled with action planning/follow-up and problem solving training [10].

It would be of interest to investigate further if this patient subgroup is the key driver of the HbA_1c_ improvements in our study, and whether the improvements achieved will sustain or increase over time.

### LDL-C and BP Reductions after PEP

Structured lifestyle education should aim at the control of all cardiovascular risk factors including lipids and blood pressure, in addition to improving glycemic control [12], [28], [37]. Our study showed that PEP improved LDL-C control, as reflected by the decreased overall mean values and increased proportion of patients with LDL-C ≤2.6 mmol/L, but no significant benefit in blood pressure control found. Other studies have also found self-management education interventions had relatively minor impacts on blood pressure control [28], [37], possibly due to the stringent targets expected for T2DM patients.

### Service Utilization Rates

Our study found some interesting changes in health service utilization rates associated with PEP participation. The PEP group had fewer GOPC visits but more SOPC visits compared with the non-PEP group. A review paper produced by the US Centre for Disease Control concluded that there was a high level of evidence to support that chronic disease self-management programmes effectively improved health utilization [38]. For diabetes-specific self-management programme, outcomes from individual randomized controlled trials had in some, but not all cases, demonstrated reduced service utilization [19], [39].

Our study showed that the effect of PEP on health service utilization can be complex. Although program success is often perceived as reduced utilization, in some cases, patients participating in PEP would be more aware of diabetic complications leading to a higher demand for referrals to specialist care. Analyses on the types of SOPC attendances (scheduled and unscheduled visits) and whether this trend would continue in the long term should be carried out to determine the true impact of PEP on health service utilization.

The relative decline in primary care consultations was three times more than the relative increase in SOPC visits, resulting in reduced overall health service utilization in PEP group. However, the additional health care expenditure incurred by increased SOPC visits may outweigh the savings from reduced GOPC visits, and therefore it remains uncertain whether PEP is cost-effective or even cost-saving. To address this question, an in-depth cost-effectiveness analysis of PEP versus usual care is currently underway, taking into account both the clinical improvements and health service costs over the phases of setup and operation.

### Limitations

Our study had three limitations. First, patients participated in the study might be those who were more motivated and proactive in seeking support. Second, some patients in the PEP group might be receiving co-interventions, for example multi-disciplinary risk assessment and management programme (RAMP) [40], in addition to PEP during the study period. Third, the control subjects might not be matched to cases by all potential confounders. It cannot be excluded that some control subjects were in secondary care.

## Conclusions

Our study provided evidence in support of the value of structured group-based empowerment programs for T2DM patients in the primary-care setting. Different from most other studies on self-management programs, this evaluation study investigated the impacts of PEP intervention in the ‘real-world setting’. The strength of our study lies in the large number and diversity of patients included. Our study also showed that it is feasible to program integrate medical and NGO services in the community to improve the quality of diabetes care. Given the improvements in metabolic control associated with PEP, further studies are warranted to evaluate whether these benefits, collectively, would translate into a reduction in the overall cardiovascular risk and other diabetes complications.

## Supporting Information

Table S1 **Adjusted Analysis on the Effects of PEP Implementation on the Clinical and Health Service Utilization Outcomes by Generalized Estimating Equation.**
(PDF)

Checklist S1 **Supporting TREND Checklist.**
(PDF)

Protocol S1 **Study Protocol.**
(DOC)
