# Supplementary material for: Effects of Patient Empowerment Programme (PEP) on Clinical Outcomes and Health Service Utilization in Type 2 Diabetes Mellitus in Primary Care: An Observational Matched Cohort Study
Source: PLoS One. 2014 May 1;9(5):e95328. doi: 10.1371/journal.pone.0095328 (PMC4006782; doi:10.1371/journal.pone.0095328)
Supplement: Table S1 — Adjusted Analysis on the Effects of PEP Implementation on the Clinical and Health Service Utilization Outcomes by Generalized Estimating Equation. (PDF) [file pone.0095328.s001.pdf]

Table S1. Adjusted Analysis on the Effects of PEP Implementation on the Clinical and Health Service Utilization Outcomes by Generalized Estimating Equation

	Generalized Estimating Equation						Adjusted Difference-in-difference		
	PEP		Time at 12 months follow-up		PEP * 12 months follow-up				
	Coefficient	P-value	Coefficient	P-value	Coefficient	P-value	Estimate	95%CI	P-value†
Linear identity link									
HbA1c	0.052 (-0.044,0.147)	0.288	-0.037 (-0.117,0.043)	0.369	-0.160 (-0.272,-0.048)	0.005*	-0.160	(-0.272,-0.048)	0.005*
SBP	-0.971 (-2.364,0.423)	0.172	-1.981 (-3.100,-0.862)	0.001*	-2.127 (-3.702,-0.553)	0.008*	-2.127	(-3.702,-0.553)	0.008*
DBP	0.538 (-0.324,1.400)	0.221	-1.258 (-1.876,-0.640)	<0.001*	-1.512 (-2.382,-0.643)	0.001*	-1.512	(-2.382,-0.643)	0.001*
LDL-C	-0.089 (-0.160,-0.017)	0.016*	-0.135 (-0.198,-0.073)	<0.001*	-0.129 (-0.212,-0.045)	0.002*	-0.129	(-0.212,-0.045)	0.002*
Binary logistic link									
HbA1c ≤7%	0.000 (-0.164,0.164)	1.000	0.014 (-0.128,0.156)	0.847	0.205 (0.007,0.404)	0.043*	5.100%	(0.002,0.100)	0.043*
SBP ≤130mmHg	0.121 (-0.051,0.292)	0.169	0.211 (0.059,0.363)	0.007*	0.122 (-0.090,0.335)	0.259	3.136%	(-0.021,0.084)	0.240
DBP ≤80mmHg	0.028 (-0.152,0.209)	0.758	0.231 (0.077,0.385)	0.003*	0.198 (-0.023,0.418)	0.079	3.675%	(-0.008,0.081)	0.107
SBP/DBP≤130/80mmHg	0.126 (-0.050,0.302)	0.161	0.245 (0.091,0.399)	0.002*	0.070 (-0.145,0.285)	0.525	1.881%	(-0.032,0.070)	0.471
LDL-C ≤2.6mmol/L	0.185 (-0.007,0.377)	0.059	0.322 (0.146,0.498)	<0.001*	0.310 (0.077,0.544)	0.009*	7.878%	(0.022,0.135)	0.006*
Poisson loglinear link									
GOPC	-0.091 (-0.130,-0.051)	<0.001*	0.107 (0.083,0.131)	<0.001*	-0.185 (-0.220,-0.149)	<0.001*	-0.813	(-0.968,-0.659)	<0.001*
SOPC	-0.009 (-0.066,0.049)	0.771	0.035 (0.004,0.065)	0.026*	0.088 (0.045,0.131)	<0.001*	0.195	(0.103,0.286)	<0.001*
ED	-0.182 (-0.312,-0.053)	0.006*	-0.034 (-0.138,0.069)	0.515	0.008 (-0.145,0.161)	0.921	0.005	(-0.055,0.066)	0.865
Inpatient Admission	-0.255 (-0.443,-0.067)	0.008*	0.020 (-0.130,0.170)	0.795	0.069 (-0.156,0.294)	0.546	0.011	(-0.033,0.056)	0.615

Note:

HbA1c = Haemoglobin A1c; SBP = Systolic Blood Pressure; DBP = Diastolic Blood Pressure; LDL-C = Low Density Lipoprotein – Cholesterol;

GOPC = General Outpatient Clinic; SOPC = Specialist Outpatient Clinic; ED = Emergency Department

* Statistically different (P<0.05) in coefficients

† P-value of testing significance in adjusted difference-in-difference estimate
