# Supplementary material for: Effects of Patient Empowerment Programme (PEP) on Clinical Outcomes and Health Service Utilization in Type 2 Diabetes Mellitus in Primary Care: An Observational Matched Cohort Study
Source: PLoS One. 2014 May 1;9(5):e95328. doi: 10.1371/journal.pone.0095328 (PMC4006782; doi:10.1371/journal.pone.0095328)
Supplement: Checklist S1 — Supporting TREND Checklist. (PDF) [file pone.0095328.s002.pdf]

TREND Statement Checklist

Paper Section/ Topic	Item No	Descriptor	Reported? ✓	Pg #
**Title and Abstract**				
Title and Abstract	1	• Information on how unit were allocated to interventions		abstract
		• Structured abstract recommended		abstract
		• Information on target population or study sample		abstract
**Introduction**				
Background	2	• Scientific background and explanation of rationale		Introduction
		• Theories used in designing behavioral interventions		
**Methods**				
Participants	3	• Eligibility criteria for participants, including criteria at different levels in recruitment/sampling plan (e.g., cities, clinics, subjects)		Setting of PEP
		• Method of recruitment (e.g., referral, self-selection), including the sampling method if a systematic sampling plan was implemented		Setting of PEP
		• Recruitment setting		Subject
		• Settings and locations where the data were collected		Subject
Interventions	4	• Details of the interventions intended for each study condition and how and when they were actually administered, specifically including:		
		○ Content: what was given?		Setting of PEP
		○ Delivery method: how was the content given?		Setting of PEP
		○ Unit of delivery: how were the subjects grouped during delivery?		Setting of PEP
		○ Deliverer: who delivered the intervention?		Setting of PEP
		○ Setting: where was the intervention delivered?		Setting of PEP
		○ Exposure quantity and duration: how many sessions or episodes or events were intended to be delivered? How long were they intended to last?		Setting of PEP
		○ Time span: how long was it intended to take to deliver the intervention to each unit?		Setting of PEP
		○ Activities to increase compliance or adherence (e.g., incentives)		NA
Objectives	5	• Specific objectives and hypotheses		Introduction
Outcomes	6	• Clearly defined primary and secondary outcome measures		Outcome Measures
		• Methods used to collect data and any methods used to enhance the quality of measurements		Outcome Measures
		• Information on validated instruments such as psychometric and biometric properties		NA
Sample Size	7	• How sample size was determined and, when applicable, explanation of any interim analyses and stopping rules		NA
Assignment Method	8	• Unit of assignment (the unit being assigned to study condition, e.g., individual, group, community)		Subject
		• Method used to assign units to study conditions, including details of any restriction (e.g., blocking, stratification, minimization)		Subject
		• Inclusion of aspects employed to help minimize potential bias induced due to non-randomization (e.g., matching)		Subject

TREND Statement Checklist

Blinding (masking)	9	• Whether or not participants, those administering the interventions, and those assessing the outcomes were blinded to study condition assignment; if so, statement regarding how the blinding was accomplished and how it was assessed.		NA
Unit of Analysis	10	• Description of the smallest unit that is being analyzed to assess intervention effects (e.g., individual, group, or community)		NA
		• If the unit of analysis differs from the unit of assignment, the analytical method used to account for this (e.g., adjusting the standard error estimates by the design effect or using multilevel analysis)		Data Analysis
Statistical Methods	11	• Statistical methods used to compare study groups for primary methods outcome(s), including complex methods of correlated data		Data Analysis
		• Statistical methods used for additional analyses, such as a subgroup analyses and adjusted analysis		Data Analysis
		• Methods for imputing missing data, if used		NA
		• Statistical software or programs used		Data Analysis
**Results**				
Participant flow	12	• Flow of participants through each stage of the study: enrollment, assignment, allocation, and intervention exposure, follow-up, analysis (a diagram is strongly recommended)		NA
		○ Enrollment: the numbers of participants screened for eligibility, found to be eligible or not eligible, declined to be enrolled, and enrolled in the study		Fig1
		○ Assignment: the numbers of participants assigned to a study condition		Fig1
		○ Allocation and intervention exposure: the number of participants assigned to each study condition and the number of participants who received each intervention		Fig1
		○ Follow-up: the number of participants who completed the follow-up or did not complete the follow-up (i.e., lost to follow-up), by study condition		Fig1
		○ Analysis: the number of participants included in or excluded from the main analysis, by study condition		NA
		• Description of protocol deviations from study as planned, along with reasons		NA
Recruitment	13	• Dates defining the periods of recruitment and follow-up		Subject
Baseline Data	14	• Baseline demographic and clinical characteristics of participants in each study condition		T1
		• Baseline characteristics for each study condition relevant to specific disease prevention research		T1
		• Baseline comparisons of those lost to follow-up and those retained, overall and by study condition		NA
		• Comparison between study population at baseline and target population of interest		NA
Baseline equivalence	15	• Data on study group equivalence at baseline and statistical methods used to control for baseline differences		Result

TREND Statement Checklist

Numbers analyzed	16	• Number of participants (denominator) included in each analysis for each study condition, particularly when the denominators change for different outcomes; statement of the results in absolute numbers when feasible	T2
		• Indication of whether the analysis strategy was "intention to treat" or, if not, description of how non-compliers were treated in the analyses	Data Analysis
Outcomes and estimation	17	• For each primary and secondary outcome, a summary of results for each estimation study condition, and the estimated effect size and a confidence interval to indicate the precision	Results
		• Inclusion of null and negative findings	Results
		• Inclusion of results from testing pre-specified causal pathways through which the intervention was intended to operate, if any	Results
Ancillary analyses	18	• Summary of other analyses performed, including subgroup or restricted analyses, indicating which are pre-specified or exploratory	NA
Adverse events	19	• Summary of all important adverse events or unintended effects in each study condition (including summary measures, effect size estimates, and confidence intervals)	NA
**DISCUSSION**			
Interpretation	20	• Interpretation of the results, taking into account study hypotheses, sources of potential bias, imprecision of measures, multiplicative analyses, and other limitations or weaknesses of the study	Discussion
		• Discussion of results taking into account the mechanism by which the intervention was intended to work (causal pathways) or alternative mechanisms or explanations	Discussion
		• Discussion of the success of and barriers to implementing the intervention, fidelity of implementation	Discussion
		• Discussion of research, programmatic, or policy implications	Discussion
Generalizability	21	• Generalizability (external validity) of the trial findings, taking into account the study population, the characteristics of the intervention, length of follow-up, incentives, compliance rates, specific sites/settings involved in the study, and other contextual issues	Discussion
Overall Evidence	22	• General interpretation of the results in the context of current evidence and current theory	Discussion

From: Des Jarlais, D. C., Lyles, C., Crepaz, N., & the Trend Group (2004). Improving the reporting quality of nonrandomized evaluations of behavioral and public health interventions: The TREND statement. *American Journal of Public Health*, 94, 361-366. For more information, visit: http://www.cdc.gov/trendstatement/
