# Supplementary material for: Effects of Patient Empowerment Programme (PEP) on Clinical Outcomes and Health Service Utilization in Type 2 Diabetes Mellitus in Primary Care: An Observational Matched Cohort Study
Source: PLoS One. 2014 May 1;9(5):e95328. doi: 10.1371/journal.pone.0095328 (PMC4006782; doi:10.1371/journal.pone.0095328)
Supplement: Protocol S1 — Study Protocol. (DOC) [file pone.0095328.s003.doc]

**Evaluation of Quality of Care Study Protocol**


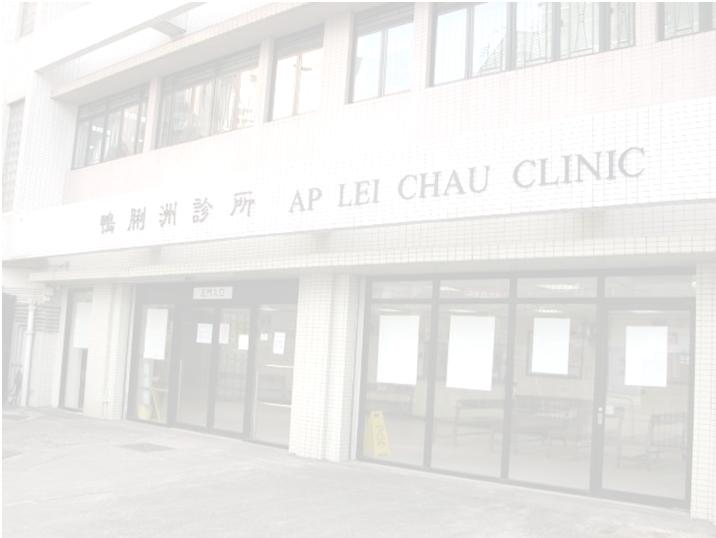
**Hospital Authority**

**Chronic Disease Management Programmes**

**Patient Empowerment Programme (PEP)**

Department of Family Medicine and Primary Care

The University of Hong Kong


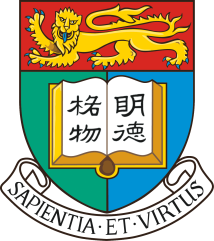

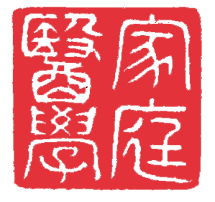
November, 2012

**Title**: Evaluation of Quality of Care – Patient Empowerment Programme, the HA

**Principal Investigator:**

Prof. Cindy L. K. Lam, Clinical Professor, Family Medicine Unit, the University of Hong Kong

**Co-investigators:**

Dr. William C. W. Wong, Clinical Associate Professor, Department of Family Medicine and Primary Care, the University of Hong Kong

Dr. Y.L. Kwok, Senior Manager, Integrated Program, the Hospital Authority

Dr. David Chao, Chief of Service, Kowloon East Cluster, the Hospital Authority

Ms. Eva L. H. Tsui, Chief Manager, Statistics & Workforce Planning, the Hospital Authority

Dr. Elegance T. P. Lam, Post-doctoral Fellow, Department of Family Medicine and Primary Care, the University of Hong Kong

**Abstract**

**Objectives:** The aim of this study is to evaluate the quality of care (QOC) of the Hospital Authority’s (HA) patient empowerment programme (PEP) programme using an evidence-based structured and comprehensive evaluation framework to identify areas for quality enhancement.

**Design, Setting and Subjects:** A longitudinal study will be conducted using the Action Learning and Audit Spiral methodologies to measure whether the target standard of care intended by the PEP programme is achieved. Each PEP participating clinic and non-government organization (NGO) will be invited to complete a structured evaluation questionnaire. The data of all patients who have enrolled into the PEP for more than one year will be included in the evaluation on the process and outcomes of care. To determine a difference of programme performance and impact between group (PEP participants and non-participants), cohort study would be adopted. A cohort includes all eligible DM patients under care of HA GOPC at 1 Sep 2009 will be followed up on the clinical outcomes including HbA1c, blood pressure, lipids, and complications rates. To evaluate the effect of PEP on patient-reported outcomes, 550 participants, as well as 550 of those who have not participated in the PEP, will be recruited from each cluster and followed up by telephone to evaluate the effect of the programme on quality of life (QOL), patient enablement, global rating of change in health condition and private service utilization at baseline, 6, 12, 36 and 60 months after enrolment. Data on the process of care will be retrieved from the HA medical records. The cost effectiveness analysis (CEA) by mathematical modeling will be carried out by using cost and clinical outcome data provided by the HA, as well as quality-of-life and private service utilization cost data collected by the telephone surveys.

**Main Outcome Measures:** The primary outcomes are the proportion of participants who have received the planned process of care and achieved the target HbA1c and blood pressure levels.

**Data Analysis:** Descriptive statistics on proportions meeting the QOC criteria will be calculated. The changes in clinical, service, and patient reported outcomes at 6 and 12 months will assessed by paired sample t-test for each audit cycle. The audit cycle will be repeated annually over a period of 5 years.

**Results:** The QOC of the PEP programme will be determined. Areas of deficiency and possible areas for quality enhancement will be identified.

**Conclusion:** The results of this study will provide empirical evidence on whether the HA’s PEP programme can enhance QOC for patients with diabetes mellitus (DM) or hypertension (HT). The information will be used to guide service planning and policy decision making.

# Background

Diabetes mellitus (DM) and hypertension (HT) are major causes of morbidity and among the top 10 causes of deaths in Hong Kong in 2008 (Department of Health 2009). The Hospital Authority (HA) has initiated service improvement through introducing the patient empowerment programme (PEP) to improve the quality of care (QOC) for DM and HT patients. The evaluation on the QOC is an essential part of the programme in order to inform future policy. The Family Medicine Unit (FMU) of the University of Hong Kong (HKU) has been appointed by the HA to carry out the evaluation of the QOC of the programme.

The PEP programme invites non-Government organizations (NGO) with experience in community medical service and education to deliver empowerment sessions to enhance patients’ disease specific knowledge and self-management skills, self-efficacy and lifestyle modification. Eligible patients who are ambulatory, have stable mental and emotional conditions, and follow up at general outpatients clinics (GOPC) and family medicine specialist clinics (FMSC) regularly will be recruited for the PEP programme. Enrolled patients will receive generic and disease specific PEP sessions provided by the NGO. Patients will be excluded if they have severe heart failure, end stage renal failure (ESRF) or advanced eye diseases, are unable to understand or communicate in Chinese language, or refuse to give consent.

Studies have found that patient empowerment programmes are effective, especially those that include active patient involvement (Lorig and Holman 2003), in improving outcomes and are somewhat cost-effective. Although more studies would be needed to determine long-term benefits and the cost-effectiveness in both hard and soft indicators (i.e. health care utilization costs vs. less tangible measures of outcome, including quality of life (QOL) and self efficacy. Two trials in Hong Kong indicated the empowerment approach was applicable to the Hong Kong Chinese population (Chan, Chan et al. 2007; Siu, Chan et al. 2007).

## Aim of Study

The aim of this study is to evaluate the quality of care (QOC) of the PEP programme using a structured and comprehensive evaluation framework that is evidence based, and to identify areas for quality enhancement.

## Hypotheses

The following hypotheses will be tested:

1. The criteria on structure and process should be achieved by all participating clinics and NGO;
2. More patients with DM will achieve the target HbA1c after 12, 24, 36 and 48 months in the PEP programme;
3. More patients with HT or DM will achieve the target blood pressure levels after 12, 24, 36 and 48 months in the PEP programme;
4. In longer terms, HT/ DM patients will have reduced cardiovascular risks and fewer complications;
5. Patients should report an improvement in quality of life (QOL), enablement and overall health condition after the PEP programme;
6. Patients who take part in PEP programme should not have any increase in service utilization rates.

# Methods

The Action Learning (Revans 1980) and Audit Spiral methodologies (Fraser, Lakhani et al. 1998) will be used to carry out a systematic analysis of the QOC and to identify areas for enhancement in the PEP programme. Donabedian’s taxonomy of QOC on structure, processes and outcomes will be used as the evaluation framework (Donabedian 2005).

Investigators will work together with the PEP programme team to:

1. review and identify the structure process and outcome indicators;
2. define the criterion and set the target standard for each indicator;
3. identify any on-site problems of implementation of the programme;
4. provide interim feedback on QOC of the programme;
5. Identify possible actions for improvements;
6. compare the final results against set standards;
7. make recommendations for future PEP programmes.

A QOC framework will be developed by an iterative process and reconciliation between the investigators and the programme providers. This framework lists out the indicators of the structure (staff, facilities, organization, and management), process (what, when and how care is delivered), and outcomes (clinical outcomes, service utilization and patient reported outcomes) with the required criteria and standard of care to be achieved.

## Subjects

All patients who have been enrolled into the PEP programme will be included in the evaluation on process of care for each audit cycle; whereas all enrolled subjects who have been recruited for more than 12 months in the programme will be included in the evaluation on the clinical outcomes of care for each audit cycle.

To determine a difference of programme performance and impact between group (PEP participants and non-participants). A cohort of all eligible DM patients under care of HA GOPC at 1 Sep 2009 would be included for the monitoring of the changes in clinical and service utilization outcomes from Sep 2009 to Sept 2015.

550 patients who have enrolled into the PEP and 550 who have not taken part in the PEP will be invited in person from the general outpatient clinics by trained research assistants at the beginning of each evaluation cycle to take part in a telephone survey on patient reported outcomes for each cluster and written consent will be obtained (appendix A). All subjects who have given consent to the telephone follow-up survey will be interviewed by telephone within four weeks, at 6, 12, 36 and 60 months from recruitment to answer the SF-12 Health Survey on quality of life (QOL), the patient enablement Instrument, the Global Rating Scale (GRS) change in health, and a structured questionnaire on private service utilization rates.

## Sample size calculation

The sample size for the evaluation on QOL change is estimated to detect a minimally clinically important difference (MCID) in health-related quality of life (HRQOL) studies, which is equivalent to Cohen’s small effect size of 0.3 (Cohen 1988). A sample size of 350 patients in total (175 PEP participants and 175 non-participants) is needed in order to have 80% power and 95% confidence interval estimated by independent t-test to detect a small effect size (Erdfelder, Faul et al. 1996). Therefore, 1100 patients (550 PEP participants and 550 non-participants) will need to be recruited to account for 25% dropouts each follow-up (4 times).

## Data Collection

Evaluation on structure and process

The co-ordinator of each participating clinic and all participating NGO will be asked to complete the structure of care questionnaire (appendix B). Anonymized data will be retrieved from the computerized medical record system (CMS) by the HA Statistics team to determine the patient recruitment rate, enrollment rate, attendance rate, compliance with assessment as per protocol, number of investigations and referral rate.

Evaluation on outcomes of care

Anonymized data on HbA1c, blood pressure (BP), low-density lipoprotein (LDL), body mass index (BMI) and annual attendance rates for GOPC, specialist outpatient clinic (SOPC) , accident and emergency (A&E) and hospital attendance rates, patient knowledge on disease at baseline and 12 months will be retrieved from the CMS by the HA Statistics team. The audit cycle will be repeated annually over a period of 5 years. This data will be extracted are shown in the data collection form in appendix C.

The 550 patients in the PEP programme who are willing to take part in the telephone survey will be contacted by a trained research assistant who will obtain verbal consent from each patient. Each patient will then answer the Chinese (Hong Kong) Short Form-12 version 2 (SF-12v2) Health Survey within one month from enrolment, and then at 6, 12, 36 and 60 months to repeat the SF-12v2 together with the Patient Enablement Instrument (PEI), Global Rating Scale (GRS) of change in health condition and private service utilization (appendix D).

- 1. The Chinese (Hong Kong) Short Form-12 version 2 (SF-12v2) Health Survey is a generic measure HR QOL. It has been validated and normed on the general Chinese population in Hong Kong (Lam, Wong et al. 2010). It measures eight domains on physical functioning, role physical, bodily pain, general health, vitality, social functioning, role emotional and mental health on a scale range from 0 to 100. A higher score indicates better HRQOL. The eight domain scores can be summarized into two summary scores, the physical (PCS) and mental (MCS) component summary.
  2. The Patient Enablement Instrument (PEI) is a measure of patient’s enablement in coping with the illness and life (Howie, Heaney et al. 1998). It has 6 items each rated on a 3-point (0, 1, and 2) scale. The summation of the item scores gives the PEI score with a higher score indicating better enablement. The PEI has been translated into Chinese and shown to be valid and reliable in the general Chinese population (Lam, Yuen et al. 2010).
  3. The Global Rating Scale (GRS) is adapted from those used in studies by Jaeschke and Osoba et al (Jaeschke, Singer et al. 1989; Osoba, Rodrigues et al. 1998). It assesses the subject’s subject global perception of any change in the overall health condition on a 7-point scale (-3, -2, -1, 0, 1, 2, and 3) over the six months.

## Outcome measures

**Primary**

- The proportion of clinics/NGO that have satisfied each of the set structure criteria.
- The proportion of patients who have complied with the criterion process of care.
- The proportion of DM patients who have achieved a HbA1c level <7%.
- The proportion of HT/ DM patients who have achieved the target blood pressure.
- The estimated 10-year cardiovascular disease risk.

**Secondary**

- Clinical outcomes including LDL and BMI.
- Cardiovascular complications.
- Renal complications.
- Patient reported outcomes (PRO) measured by the change in SF-12v2 scores, the PEI and GRS scores at 6, 12, 36 and 60 months, and change in patient’s knowledge on disease at 12 months.
- GOPC consultation, SOPC, A&E and hospital attendance rates in the past 12 months; the audit cycle will be repeated annually over a period of 5 years.

## Data analysis

1. Descriptive statistics on standard of care will be calculated, including percentage of centers meeting each structure criterion, percentage of subjects enrolled, dropped out, completion of the programme, receiving criterion process, investigations and referral per protocol, and percentage of subjects achieving the criterion outcome.
2. The change in clinical, service, and patient reported outcomes after the PEP programme at baseline, 6, 12, 36 and 60 months will be compared by paired sample t-test. The audit cycle will be repeated annually over a period of 5 years.
3. Independent sample t-test or Chi square test as appropriate will be used to compare the clinical and service utilization outcomes between PEP patients and controls.
4. Independent sample t-test or Chi square test as appropriate will be used to compare the clinical and service utilization outcomes between results in achieved in different audit cycles.
5. Cost effectiveness analysis (CEA) by mathematical modeling will be carried out by using cost and clinical outcome data provided by the HA, as well as quality-of-life and private service utilization cost data collected by the telephone surveys.

***Feedback***

The full analysis on the standards of care will be carried out at 15, 30 and 45 months from the start of the programme to evaluate whether the set standards of care on structure, process and outcome have been achieved. The results of each evaluation on the performance of the programme will be fed back to the programme team to identify changes in quality criteria and standards and quality enhancement strategies to be implemented.

Timeline of Study

| **Dates** | **Tasks** |
| --- | --- |
| Apr 2010 – Jun 2010 | - Recruitment of research assistants - Literature review - Development of the preliminary QOC evaluation framework |
| Jul 2010 – Sep 2010 | - Refine the QOC evaluation framework - IRB application - Training of interviewers |
| Oct 2010 – Jan 2011 | - Collection of data on structure from each participating clinic and NGO - Retrieval of data on process of care, and clinical and service outcomes from HA records - Collection of data on PRO (by telephone interview) |
| Apr 2011 – Jun -2011 | - Collection of data on PRO (by follow-up telephone interview) - Interim analysis of QOC data - Feedback of interim analysis results to PEP programme team - Implementation of quality enhancement strategies if needed |
| Sep 2011 – Oct 2014 | - Retrieval of data on process of care, and clinical and service outcomes from HA records - Four cylces of data analysis on QOC of the programme - Feedback on results on QOC to PEP programme team - Implementation of quality enhancement strategies if needed |
| Nov 2014 – Apr 2015 | - Prepare final report for submission to the HA - Preparation of manuscripts for publication |

QOC = Quality of Care; PRO = Patient reported outcomes.

# Implications

The results can:

1. provide empirical data on the QOC of PEP provided by the contractor (NGO) of HA;
2. provide evidence on whether the PEP can enhance patients on self-efficacy and knowledge of their diseases;
3. provide information on the effectiveness of PEP in improving the outcome of care for DM and HT patients, in order to guide policy decision making.

# Compliance with the ICH-GCP

This protocol is complied with the ICH-GCP.

# Reference

# List of additional materials:-

- Appendix A: Written consent form to patients on the evaluation of programme
- Appendix B: Structure of care questionnaire
- Appendix C: Data collection form for extraction of data from the HA medical records
- Appendix D: Questionnaire for baseline & follow-up telephone surveys

## Appendix A: Written Consent Form to patients on the evaluation of programme

**Research conducted by**

**Department of Family Medicine and Primary Care, HKU
and the Hospital Authority**

**Information and Consent Form**

Title: Evaluation of Quality of Care – Patient Empowerment Programme

Investigators: Professor Cindy L.K. Lam, Dr. William C.W. Wong, Dr. Y.L. Kwok, Dr. Y.K. Yiu, Ms. Eva L.H. Tsui, Dr. Elegance T.P. Lam

Thank you for reading the information and agreeing to consider taking part in this study. Please read this form, and if you agree, please sign and date at the end of this Consent Form. You and your legally representatives will be updated timely of new information that may be relevant to your willingness to continue participation in study. You will have a copy of signed informed consent form.

**Study Information**

This research is a collaboration study between HKU and Hospital Authority (KWC). The aim of this research is to evaluate the health of diabetic patients and their quality of life. 550 PEP participants and 550 controls are expected to be recruited to evaluate the effect of the programme. Approximately 80 PEP participants and 80 controls will be involved in this study in KWC. You will be asked to answer a structured questionnaire by telephone interview within 2 weeks, at 6, 12, 36 and 60 months from the day of recruitment. Each telephone interview will last only 10 to 15 minutes.

If you kindly agree to take part in the study, we will ask you to provide your name and telephone number to trained interviewers of the Department of Family Medicine and Primary Care, the University of Hong Kong, who will call you by telephone. All the data collected from you will be kept confidential and no individual identity information will be disclosed in any reports, data record forms or publications. If you decide to take part in this research, please sign and date this Consent Form to indicate that you have understood the purpose and procedure of the study and you are willing to take part.

You can withdraw from the study anytime you want without infringement on any of your rights to treatment in this clinic or other services provided by the Hospital Authority.

**For further information please contact**:

Dr. Wendy Wong

Department of Family Medicine and Primary Care,

the University of Hong Kong

3/F., Ap Lei Chau Clinic, 161 Main Street, Ap Lei Chau, Hong Kong

Telephone: 2552 5756 ; Fax: 2814 7475

**Declaration on Protection of Personal Data**

Under the laws of the Hong Kong Special Administrative Region and, in particular, the Personal Data (Privacy) Ordinance, Cap 486, you enjoy or may enjoy rights for the protection of the confidentiality of your personal data, such as those regarding the collection, custody, retention, management, control, use (including analysis or comparison), transfer in or out of Hong Kong, non-disclosure, erasure and/or in any way dealing with or disposing of any of your personal data in or for this study.

By signing and dating this Consent Form, you agree to allow the collection, custody, retention, management, control, and use your personal data in this study in ways described in the Information Leaflet. For any query, you should consult the Privacy Commissioner for Privacy Data or his office ( Tel No. 2827 2827 ) as to the proper monitoring or supervision of your personal data protection so that your full awareness and understanding of the significance of compliance with the law governing privacy data is assured.

**Title: Evaluation of Quality of Care – Patient Empowerment Programme**

**Consent**

The following statements are to check that you understand and consent to the procedures involved in taking part in this research:

1. I confirm that I have read and understood (or had someone read and explained) the information for the above study and have been given a copy to keep. I have had the opportunity to ask questions about the project and I understand why the research is being done and any risks involved.
2. I understand that my participation is voluntary.
3. I agree to take part in the study.
4. I am happy for the research team to contact me by telephone.
5. I agree to allow the research team to obtain from my doctors and the Hospital Authority anonymous clinical data extracted from my medical record for the purpose of the study.
6. I understand that all information that I provide to the research team will be kept confidential and only the investigators and their research team will have access to it.
7. I understand that direct access to my original medical records is granted to monitors, auditors, KWC-REC and other regulatory authorities to verify clinical trial procedure and / or data without violating my confidentiality to the extent permitted by applicable laws and regulations, and by signing the written informed consent form, I or my legally authorized representatives are authorizing such access.
8. I understand that my record confidentiality and use to the extent permitted by applicable laws and regulations will be safeguarded (if the study results are published, my identity will remain confidential).
9. I understand how the data will be collected, that giving data for this research is voluntary and that I am free to withdraw the permission to use my data at any time, without giving reason and without my medical treatment or legal rights being affected.
10. I understand that I will need to have five telephone interviews in 2 weeks, at 6, 12, 36 and 60 months time.
11. I understand that I will not benefit financially from this research.
12. I understand that I will not benefit clinically from this research.
13. I understand that there are no known risks to participating in this research.
14. I understand that I am free to withdraw from the study at any time, without giving reason and without my medical treatment or legal rights being affected in any way.
15. I understand the investigators have the right to exclude me from the study in the event of inter-current illness, adverse event, protocol violations, or other reasons.

**Please sign and date this Consent Form below:**

………………………………………… ……………………………….. …..………………

Name of Subject in BLOCK letters Signature Date

………………………………………… ……………………………….. …..………………

Name of Investigator in BLOCK letters Signature Date

………………………………………… ……………………………….. …..………………

Name of Witness in BLOCK letters Signature Date

**香港大學家庭醫學及基層醫療學系**

**及醫院管理局**

**參與研究資料及同意書**

**《病人自強計劃》**

**研究人員: 林露娟教授, 黃志威醫生, 郭旭龍醫生, 姚玉筠醫生, 徐麗卿小姐, 林定珮博士**

謝謝您閱讀這項研究的資料和同意考慮參加。請細閱此同意書，如您同意的話，請在此同意書末端簽署和寫上日期。如若有新資料或會影響您繼續參與這項研究的意願，我們(香港大學家庭醫學及基層醫療學系)將會提供以上最新信息給您和您的法定代表。您將會得到一份已簽署的同意書作記錄之用。

**研究資料**

這是香港大學及醫院管理局(九龍西聯網)的共同研究。這研究的目的是評估糖尿病病人的健康及生活質素。另外，為找出《病人自強計劃》的成效，需要徵募五百五十位糖尿病《病人自強計劃》參加者及五百五十位研究對照對象作比較之用。研究小組會在九龍西聯網徵募大約八十位參加者及八十位研究對照。參與者會於招募日起兩個星期內、六個月、十二個月、三十六個月及六十個月後接受共五次的接受電話訪問，每次歷時約十至十五分鐘。

如果您同意參加這項研究，請您提供您的姓名及電話。我們(香港大學家庭醫學及基層醫療學系)的訪問員會致電話訪問您。您所提供的資料將會受到研究小組的絶對保密，任何個人資料將不會在研究報告中披露。如果您決定參與這項研究，請您在這份同意書末端簽署和寫上日期來表明您自願參加和明白研究內容及整個研究程序。

您有權隨時退出這項研究，您的退出不會影響您在該診所的治療或使用其他醫院管理局所提供的醫療服務。

**如您對此項研究有疑問，請聯絡：**

香港大學聯絡：

鴨脷洲大街161號鴨脷洲診所三樓

香港大學家庭醫學及基層醫療學系 黃韻婷博士

電話：2552 5756；傳真號碼：2814 7475

**個人資料保障權聲明**

依照香港特別行政區的法律，個人資料(私隱)條例(第486章)，您享有個人資料保密的保障權，例如有關在此研究中收集、持有、保存、處理、控制，或使用(包括分析或比較)，在香港以內或以外的傳送，不作披露，删除和/或使用任何途徑去處理或棄置您的任何個人資料。

您在此同意書簽署和寫上日期，表示您同意容許我們在此研究中收集、持有、保存、處理、控制，或使用您的個人資料作為在資料小册子所述目的之用。如您有任何疑問，請諮詢私隱專員公署或其辨公室 (電話: 2827 2827 )有關保障您個人資料的適當監管或指導，以確保您完全知道和明白遵守個人資料(私隱)條例的重要。

**《病人自強計劃》**

**同意書**

以下句子是為了確定您是否明白及同意參與這項研究的各程序：

1. 我肯定我已閱讀過和明白(或由他人讀出並解釋) 有關以上研究的資料，並得到一份副本作保存。我有機會對這研究計劃提出疑問，並了解進行這研究的原因和所涉及的風險。
2. 我明白我的參與純屬自願。
3. 我同意參與這項研究。
4. 我樂意給研究小組提供我的姓名及聯絡電話，以便於稍後時間進行電話訪問。
5. 我同意讓研究小組向我的醫生和醫管局索取我不記名的病歷資料以供研究分析。
6. 我明白所有我自己提供給研究小組的資料將會絶對保密，只有研究人員及他們的研究小組能夠閱看。
7. 我明白我只授權於監測員、審計員、九龍西聯網臨床研究倫理委員會和其他監管機構直接查閱我的正本病歷記錄，以驗證臨床測試程序及/或數據，而又不違反適用的法律和法規允許的範圍內保密我的資料，並通過簽署書面的同意書，我或我的合法授權代表授權這種資料查閱。
8. 我明白我自己的個人資料，將依照現行法律保密 (如研究結果公開，我的身份將依然保密)。
9. 我明白這計劃收集資料的方法，並自願地為這研究提供此資料。我有權隨時拒絕提供資料或收回這些資料的使用權，而不用提供任何理由，以及不會影響到我的治療和法律權利。
10. 我明白我將需要在參加計劃後的兩星期內、六個月、十二個月、三十六個月及六十個月後接受共五次的電話訪問。
11. 我明白自己將不會得到任何金錢利益。
12. 我明白自己將不會得到任何醫療上的利益。
13. 參與是項研究並不會構成任何已知的風險。
14. 我明白我有權隨時退出這項研究，而不用提供任何理由，以及不會影響到我的治療和法律權利。
15. 我明白研究人員有權終止我參與此研究，假如出現拼發症或不良反應、或未能符合計劃書要求或其他原因。

請在此同意書末端簽署和寫上日期:

|  |  |  |  |  |
| --- | --- | --- | --- | --- |
| 參加者姓名 (請用正楷寫上) |  | 簽署 |  | 日期 |
|  |  |  |  |  |
| 研究員姓名 (請用正楷寫上) |  | 簽署 |  | 日期 |
|  |  |  |  |  |
| 見證人姓名 (請用正楷寫上) |  | 簽署 |  | 日期 |

## Appendix B: Structure of care questionnaire

| **PEP Structure of Care Questionnaire** | | | |
| --- | --- | --- | --- |
| **Please indicate whether the following statement is applicable for your clinic.** | | | |
|  | | | |
| 1 | There **must** be at least 1 registered nurse, 1 healthcare professional and 1 clerical staff in the participating NGO. | Yes | No |
|  |  |  |  |
| 2 | The in-charge staff in each NGO **should** be familiar with the programme objectives and logistics. | Yes | No |
|  |  |  |  |
| 3 | The in-charge staff in NGO **should** know the recruitment, enrolment and programme protocol. | Yes | No |
|  |  |  |  |
| 4 | The in-charge staff in Cluster Programme Office **should** have undergone PPi-PEP and e-Referral training for this programme. | Yes | No |
|  |  |  |  |
| 5 | Specific data collection forms (electronic) **must** be used to collect patient data for this programme. | Yes | No |
|
|  |  |  |  |
| 6 | Clinical staff **must** have access to the e-referral system for patient referral. | Yes | No |
|
|  |  |  |  |
| 7 | NGO staff **should** have access to the PPI-PEP system for patient data collection and retrieval. | Yes | No |
|  |  |  |  |
| 8 | Facilities for patient recruitment and enrollment **should** be available in the clinic. | Yes | No |
|  |  |  |  |
| 9 | Toolkits on generic self-efficacy enhancement and lifestyle modification component **should** be available | Yes | No |
|
|  |  |  |  |
| 10 | Toolkits on specific disease knowledge on DM and HT **should** be available in NGO | Yes | No |
|  |  |  |  |
| 11 | There **must** be a record of patients referred in the PPI-PEP system shared by authorized members of the programme. | Yes | No |
|  |  |  |  |
| 12 | There **should** be a record of enrolled patients in the PPI-PEP system shared by authorized members of the programme. | Yes | No |
|  |  |  |  |
| 13 | The patients’ doctor **should** be able to access the information that the patient has enrolled to the PEP. | Yes | No |
|  |  |  |  |
| 14 | After completion of the programme, the end-of-programme summary **could** be available for the patient to their doctors. | Yes | No |
|  |  |  |  |
| 15 | There **should** be meetings among staff of the participating NGO to monitor the performance of the programme. | Yes | No |

## Appendix C: Data collection form for extraction of data from the HA medical records

| **Data Required** | **Number of  patients** | **Time (in days) from recruitment** |
| --- | --- | --- |
|
| Uptake of programme* | | |
| Patients identified eligible |  | NA |
| Patients recruited |  | NA |
| Patients enrolled |  |  |
| Process of care* | | |
| Patients attended at least 1 generic session |  |  |
| Patients attended at least 1 disease specific session |  |  |
| Patients completed the programme |  |  |
| Patients dropped out or discharged from programme |  |  |
| Patients identified to have problems (e.g. poor drug compliance, overweight, foot deformity, smoking) |  |  |

*include only subjects who have enrolled more than 12 months

**Patients require special treatment:**

| **Data Required** | **Number of  patients** | **Time (in days) from problem recognition** |
| --- | --- | --- |
|
| Identified to have problem being given treatments per protocol |  |  |
| Referred for additional service |  |  |
| Refused additional service |  |  |

| **Time (Month)** | **Data required** |
| --- | --- |
| 0, 12, 24, 36 and 48 | **Clinical data** |
|  | HbA1c |
|  | Systolic BP |
|  | Diastolic BP |
|  | BMI |
|  | LDL-C |
|  | **Service utilization** (in the past 12 months) |
|  | Number of hospitalization |
|  | Number of A&E attendance |
|  | Number of GOPC consultation |
|  | Number of SOPC consultation |
| 0 and 6 months | **Patient reported** |
|  | Disease knowledge score |
|  | QoL score |
|  | PEI* |
|  | GRS* |

BP = Blood Pressure; BMI = Body Mass Index; LDL-C = Low density Lipoprotein Cholesterol; GOPC = General Outpatient Clinic; SOPC = Specialist Outpatient Clinic; KAP = Knowledge, Attitude and Practice.

*Measured at 6 months only.

## Appendix D: Questionnaires for baseline & follow-up telephone surveys

**《評估慢性疾病管理計劃成效**

**–病人自强計劃》**

**Baseline Evaluation**

您好！＿＿先生/小姐。我係香港大學家庭醫學及基層醫療學系嘅訪問員___________（請報自己姓名）。我地替醫院管理局來評估**病人自强計劃**對你既成效。我將會請您回答一些問題，阻您大約十至十五分鐘時間；另外，六個月及十二個月後將會再次打電話來跟進您既情況。

所有個人資料係會絕對保密。（如您對是項研究有疑問，歡迎起辦公時間打電話2552-5756到香港大學家庭醫學及基層醫療學系林博士查詢。）好多謝您嘅幫忙同合作。

|  |  |
| --- | --- |
|  |  |
|  |  |
|  |  |
|  |  |
|  |  |
| **訪問日期:** |  |
|  |  |
| **訪問員編號:** |  |

**標準十二題簡明健康狀況調查表-第**二**版**

**說明：這項調查是詢問您對自己健康狀況的了解。此項資料記錄您的自我感覺和日常生活的情況。**

**請在一個方格內填上X號來回答每個問題。如果您不肯定怎樣回答，請按照您的理解選擇最合適的答案。**

**1. 總括來說，您認為您的健康狀況是：**

| 極好 | | 很好 | | 好 | | 一般 | | 差 |
| --- | --- | --- | --- | --- | --- | --- | --- | --- |
| 1 | 2 | | 3 | | 4 | | 5 | |

**2. 下列問題是您日常生活中可能進行的活動。以您目前的健康狀況，您在進行這些活動時，有沒有受到限制？如果有的話，程度如何？**

|  | 有很大限制 | 有一點限制 | 沒有任何限制 |
| --- | --- | --- | --- |
| a. 中等強度的活動，比如搬桌子，使用吸塵器清潔地面，玩保齡球或打太極拳 | 1 | 2 | 3 |
| b. 上幾層樓梯 | 1 | 2 | 3 |

**3. 在過去四個星期裏，您在工作或其它日常活動中，有多少時間會因為身體健康的原因而遇到下列的問題？**

|  | 常常如此 | 大部分時間 | 有時 | 偶爾 | 從來沒有 |
| --- | --- | --- | --- | --- | --- |
| a. 實際做完的比想做的要少 | 1 | 2 | 3 | 4 | 5 |
| b. 工作或其它活動的種類受到限制 | 1 | 2 | 3 | 4 | 5 |

**4. 在過去的四個星期裏，您在工作或其它日常活動中，有多少時間由於情緒方面的原因（比如感到沮喪或焦慮）遇到下列的問題？**

|  | 常常如此 | 大部分時間 | 有時 | 偶爾 | 從來沒有 |
| --- | --- | --- | --- | --- | --- |
| a. 實際做完的比想做的要少 | 1 | 2 | 3 | 4 | 5 |
| b. 工作時或從事其它活動時不如往常細心了 | 1 | 2 | 3 | 4 | 5 |

**5. 在過去四個星期裏，您身體上的疼痛對您的日常工作（包括上班和家務）有多大影響？**

| 毫無影響 | | 有很少影響 | 有一些影響 | | 有較大影響 | | 有極大影響 |
| --- | --- | --- | --- | --- | --- | --- | --- |
| 1 | 2 | | 3 | 4 | | 5 | |

**6. 下列問題是有關您在過去四個星期裏您覺得怎樣和您其它的情況。針對每一個問題，請選擇一個最接近您的感覺的答案。在過去四個星期裏有多少時間：**

|  | 常常如此 | 大部分時間 | 有時 | 偶爾 | 從來沒有 |
| --- | --- | --- | --- | --- | --- |
| a. 您感到心平氣和？ | 1 | 2 | 3 | 4 | 5 |
| b. 您感到精力充足？ | 1 | 2 | 3 | 4 | 5 |
| c. 您覺得心情不好，悶悶不樂？ | 1 | 2 | 3 | 4 | 5 |

**7. 在過去四個星期裏，有多少時間由於您的身體健康或情緒問題妨礙了您的社交活動 (比如探親、訪友等）？**

| 常常有妨礙 | | 大部分時間有妨礙 | | 有時有妨礙 | | 偶爾有妨礙 | | 完全沒有妨礙 |
| --- | --- | --- | --- | --- | --- | --- | --- | --- |
| 1 | 2 | | 3 | | 4 | | 5 | |

多謝您參與研究！

----------------------------------------------------完-------------------------------------------------

**私家醫療服務的使用**

| 1. **在過去四個星期裏，**你有冇睇過私家醫生 (西醫 / 中醫)？  *(如答沒有, 回答問題2)* | 1.  2. | | | 有  沒有 | | | | | | | | | |
| --- | --- | --- | --- | --- | --- | --- | --- | --- | --- | --- | --- | --- | --- |
|  | 1. | 普通科 | | | | 2. | 專科 | | | 3. | 中醫 | | |
| a. 請問你睇過私家醫生幾多次？ | 1. | | | | 次 | 2. | 次 | | | 3. | 次 | | |
| b. 請問你由屋企**平均**要搭幾耐車先到私家診所呢？ | 1. | | | | 分鐘/小時 | 2. | 分鐘/小時 | | | 3. | 分鐘/小時 | | |
| c. 請問你由屋企搭車去私家診所要幾多錢呢？ | 1. | | | |  | 2. |  | | | 3. |  | | |
| d. 請問你睇一次私家醫生**平均**要幾耐？ (包括等候同埋見醫生嘅時間) | 1. | | | | 分鐘/小時 | 2. | 分鐘/小時 | | | 3. | 分鐘/小時 | | |
| e. **每次**求診嘅費用有幾多  (包括僱主或保險公司嘅補貼)？ | 1. | | | |  | 2. |  | | | 3. |  | | |
| f. 有冇人陪你睇私家醫生呢？佢係邊個？  (性別：男/女) | 1. | | i.  ii. | | 有 ( 男/女 )  沒有 | 2. | | i.  ii. | 有 ( 男/女 )  沒有 | 3. | | i.  ii. | 有 ( 男/女 )  沒有 |
| g. 佢有冇請假呢？ | 1. | | i.  ii. | | 有  沒有 | 2. | | i.  ii. | 有  沒有 | 3. | | i.  ii. | 有  沒有 |

| 2. **在過去四個星期裏，**你有冇試過自己買藥食？  *(如答沒有, 回答問題3)* | 1.  2. | 有  沒有 | |
| --- | --- | --- | --- |
| 怎樣處理，幾多次，**每次**幾多錢？ | 次數 | | 每次費用 ($) |
| 1. 西藥成藥 | 1. | | 2. |
| b. 中藥成藥 (例如：牛黃解毒片、銀翹) | 1. | | 2. |
| c. 中草藥 (藥材舖執藥) | 1. | | 2. |
| 1. 其他，請註明：______________ | 1. | | 2. |

| 3. **在過去六個月裏，**你有冇入住私家醫院？  *(如答沒有, 回答問題4)* | 1.  2. | 有  沒有 |
| --- | --- | --- |
| a. 請問你留左幾多日院呢？ | 日 | |
| b. 請問你由屋企**平均**要搭幾耐車先到私家醫院呢？ | 分鐘/小時 | |
| c. 請問你由屋企搭車去私家醫院要幾多錢呢？ | $ | |
| d. **上一次**入住醫院嘅費用有幾多  (包括僱主或保險公司嘅補貼)？ | $ | |
| e. 有冇人係你**上一次**入院時照顧患病嘅你？佢係邊個？ (性別：男/女) | 1.  2. | 有 _____ (男/女)  沒有 |
| f. 係你**上一次**入院時佢請咗幾多日假照顧你？ | 日 | |

**家居照顧**

| 4. **在過去六個月裏，**當你患病嘅時候有冇人照顧你嘅起居飲食？佢係邊個？ (性別：男/女) | 1.  2. | 有 _____ (男/女)  沒有 | | |
| --- | --- | --- | --- | --- |
| 4a. 佢洗唔洗請假照顧你？唔番工定係唔番學？如果要請假，請幾多日呢？ | 1. | 有 | i.  ii. | 唔番工________日  唔番學 ________日 |
|  | 2. | 沒有 | | |

**生活方式**

吸煙

| 5. 你有冇食煙嘅習慣？*(如答沒有, 回答問題****5e****)* | 1.  2. | | 有  沒有 | | | | |
| --- | --- | --- | --- | --- | --- | --- | --- |
| a. 請問你食煙嘅習慣是： | 1. 現在完全戒煙 | | | | | 2. 現在間中食煙，每日不夠一支 | 3. 現在每日食煙起碼一支 |
| b. 請問你已戒煙有幾耐？ | ___年___ 月 | | | | |  |  |
| c. 請問你(以前 / 現在)平均每日吸多少支煙? | 1. 支 | | | | | 2. 支 | 3. 支 |
| d. 請問你食左幾多年煙？ | 1. 年 | | | | | 2. 年 | 3. 年 |
| e. 不計算你自己，請問你**屋企**同埋**工作範圍**有沒有其他人食煙呢？ | 1. | 屋企: | | i.  ii. | 有  沒有 | | |
|  | 2. | 工作範圍: | | i.  ii. | 有  沒有 | | |

運動習慣

| 6. 你有冇定期做運動嘅習慣？  *(如答沒有, 回答問題7)* | 1.  2. | 有  沒有 | | | | |
| --- | --- | --- | --- | --- | --- | --- |
| a. 你是參與什麼類型的體育活動(你可選擇多過一個答案) | 1.有氧健身法 | | 2. 舒展運動 | 3. 平路慢步 | 4. 太極/瑜珈 | 5. 其他： _________ |
| b. 每星期做幾多次運動？ | 1. ____ 次 | | 2. _____ 次 | 3._____ 次 | 4._____ 次 | 5. _____ 次 |
| c. 你每次平均做幾耐運動？ | 1._____分鐘  a. < 15 分鐘  b. 15-30分鐘  c. > 30分鐘 | | 2._____分鐘  a. < 15 分鐘  b. 15-30分鐘  c. > 30分鐘 | 3._____分鐘  a. < 15 分鐘  b. 15-30分鐘  c. > 30分鐘 | 4._____分鐘  a. < 15 分鐘  b. 15-30分鐘  c. > 30分鐘 | 5.____分鐘  a. < 15 分鐘  b. 15-30分鐘  c. > 30分鐘 |

**個人資料**

| 7. 請問你嘅婚姻狀況係？ | 1. 單身 | | 2. 已結婚 | | | 3. 分居/離婚 | | | 4. 配偶去世 | | | | | 5. 拒絕回答 | | |
| --- | --- | --- | --- | --- | --- | --- | --- | --- | --- | --- | --- | --- | --- | --- | --- | --- |
|  |  | |  | | |  | | |  | | | |  | | | |
| 8. 請問你平均每月嘅家庭收入係 (包括所有來源)？  $________________ | 1.  少過  $2,000 | 2.  $2,000至$3,999 | | 3.  $4,000至$5,999 | 4.  $6,000至$7,999 | | | 5.  $8,000至$9,999 | | | 6.  $10,000至$14,999 | 7.  $15,000至  $19,999 | | | | 8.  $20,000至$24,999 |
|  | 9.  $25,000至$29,999 | 10.  $30,000至$39,999 | | 11.  $40,000至$59,999 | 12.  $60,000或  以上 | | | 13.  沒有收入 | | | 14.  拒絕  回答 | 15.  不知道 | | | |  |
|  |  | |  | | |  | | |  | | | |  | | | |
| 9. 請問你平均每月嘅個人收入係 (包括所有來源)？  $________________ | 1. 少過 $2,000 | | 1. $2,001   至  $5,000 | | | | 1. $5,001   至  $9,999 | | | 1. $10,000至$14,999 | | | | | 1. $15,000   至  $19,999 | |
|  | 1. $20,000 至$29,999 | | 1. $30,000或以上 | | | | 1. 沒有收入 | | | 1. 拒絕回答 | | | | | 1. 不知道 | |

----------------------------------------------------完----------------------------------------------------

**SF-12 HEALTH SURVEY (VERSION 2.0)**

INSTRUCTIONS: This survey asks for your views about your health. This information will help keep track of how you feel and how well you are able to do your usual activities.

Answer every question by marking the answer as indicated. If you are unsure about how to answer a question, please give the best answer you can.

1. In general, would you say your health is:

| Excellent | Very good | Good | Fair | Poor |
| --- | --- | --- | --- | --- |
| 1 | 2 | 3 | 4 | 5 |

2. The following questions are about activities you might do during a typical day. Does **your health now limit you** in these activities? If so, how much?

|  | Yes, limited a lot | Yes, limited a little | No, not limited at all |
| --- | --- | --- | --- |
| a. **Moderate activities**, such as moving a table, pushing a vacuum cleaner, bowling, or playing golf | 1 | 2 | 3 |
| b. Climbing **several** flights of stairs | 1 | 2 | 3 |

3. During the **past 4 weeks**, how much of the time have you had any of the following problems with your work or other regular daily activities **as a result of your physical health**?

|  | All of the time | Most of the time | Some of the time | A little of the time | None of the time |
| --- | --- | --- | --- | --- | --- |
| a. **Accomplished less** than you would like | 1 | 2 | 3 | 4 | 5 |
| b. Were limited in the **kind** of work or other activities | 1 | 2 | 3 | 4 | 5 |

4. During the **past 4 weeks**, how much of the time have you had any of the following problems with your work or other regular daily activities **as a result of any emotional problems** (such as feeling depressed or anxious)?

|  | All of the time | Most of the time | Some of the time | A little of the time | None of the time |
| --- | --- | --- | --- | --- | --- |
| a. **Accomplished less** than you would like | 1 | 2 | 3 | 4 | 5 |
| b. Did work or other activities less **carefully** than usual | 1 | 2 | 3 | 4 | 5 |

5. During the **past 4 weeks**, how much did **pain** interfere with your normal work (including both work outside the home and housework)?

| Not at all | A little bit | Moderately | Quite a bit | Extremely |
| --- | --- | --- | --- | --- |
| 1 | 2 | 3 | 4 | 5 |

6. These questions are about how you feel and how things have been with you during the **past 4 weeks**. For each question, please give the one answer that comes closest to the way you have been feeling. How much of the time during the **past 4 weeks**...

|  | All of the time | Most of the time | Some of the time | A little of the time | None of the time |
| --- | --- | --- | --- | --- | --- |
| a. Have you felt calm and peaceful? | 1 | 2 | 3 | 4 | 5 |
| b. Did you have a lot of energy? | 1 | 2 | 3 | 4 | 5 |
| c. Have you felt downhearted and depressed? | 1 | 2 | 3 | 4 | 5 |

7.During the **past 4 weeks**, how much of the time has your **physical health** or **emotional problems** interfered with your social activities (like visiting friends, relatives, etc.)?

| All of the time | Most of the time | Some of the time | A little of the time | None of the time |
| --- | --- | --- | --- | --- |
| 1 | 2 | 3 | 4 | 5 |

-------------------------------------------------end-----------------------------------------------

**Utilisation in private medical services:**

| 1. Did you have any private doctor consultation in the **last 4 weeks (western medicine / Chinese medicine)**? *(If no, go to 2)* | 1.  2. | Yes  No | | | | | | | |
| --- | --- | --- | --- | --- | --- | --- | --- | --- | --- |
|  |  | General  Practitioner | |  | Specialist | |  | Chinese Medicine | |
| a. How many visits? | 1. | visits | | 2. | visits | | 3. | visits | |
| b. How long did you spend for travelling from home to clinic **on average**? | 1. | Min/Hrs | | 2. | Min/Hrs | | 3. | Min/Hrs | |
| c. How much did you spend for travelling from home to clinic? | 1. |  | | 2. |  | | 3. |  | |
| d. How long did you stay in the clinic **on average**? (Waiting time and consultation time) | 1. | Min/Hrs | | 2. | Min/Hrs | | 3. | Min/Hrs | |
| e. How much did you pay for **each visit** (including subsidies from employer or insurance company)? | 1. |  | | 2. |  | | 3. |  | |
| f. Did any person accompany with you to go for visit(s)? Who was that person? (Gender: Male /Female) | 1. | i.  ii. | Yes (M/F) No | 2. | i.  ii. | Yes(M/F)  No | 3. | i.  ii. | Yes (M/F) No |
| g. Did she/he take time off work/ study? | 1. | i.  ii. | Yes (M/F)  No | 2. | i.  ii. | Yes (M/F)  No | 3. | i.  ii. | Yes (M/F)  No |

| 2. Did you get any self medication in the **last 4 weeks**?  *(If no, go to 3)* | 1.  2. | Yes  No | |
| --- | --- | --- | --- |
| How many times did you get self medication and how much did you pay **each time**? | No. of times | | Cost each time ($) |
| 1. Western over-the-counter medicine | 1. | | 2. |
| b. Chinese over-the-counter medicine  (eg. Chinese medical products) | 1. | | 2. |
| c. Chinese herbal medicine  (eg. herbal medicines which require boiling) | 1. | | 2. |
| 1. Others, please specify:_________________ | 1. | | 2. |

| 3. Did you have any private hospitalisation in the **last 6 months**?  *(If no, go to 4)* | 1.  2. | Yes  No |
| --- | --- | --- |
| a How many days of admission? | days | |
| b. How long did you spend for travelling from home to hospital **on average**? | Min/Hrs | |
| c. How much did you spend for travel from home to hospital? | $ | |
| d. How much did you pay for the **last admission** (including subsidies from employer or insurance company)? | $ | |
| e. Did any person visit you during your **last admission**? Who was that person?  (Gender: Male /Female) | 1.  2. | Yes ( M / F )  No |
| f. How many days did she/he take time off work because of visiting you during your **last admission**? | days | |

**Home care cost:**

| 4. Did any person take care of you at home while you’re sick in the last 6 months? If so, who was that person? (Gender: Male/Female) | 1.  2. | Yes ( M / F )  No | | |
| --- | --- | --- | --- | --- |
| 4a. Did she/ he take any time off work/ study to take care of you? If so, how many days? | 1. | Yes | i.  ii. | Work ________days  study ________days |
|  | 2. | No | | |

**Lifestyle**:

Smoking

| 5. Did/do you have a habit of smoking? *(If no, go to* ***5e****)* | 1.  2. | Yes  No | | | | |
| --- | --- | --- | --- | --- | --- | --- |
| a. Your smoking habit is: | 1. Now have quit already  (former smoker) | | | | 2. Currently smoke occasionally, fewer than one cigarette a day | 3. Currently smoke, at least one cigarette a day |
| b. How long have you quitted smoking? | _____Yr(s) ____ Mth(s) | | | |  |  |
| c. How many cigarettes (past / present) on average did/do you smoke a day? | 1. cigarettes | | | | 2. cigarettes | 3. cigarettes |
| d. How many years did you/have you smoked? | 1. years | | | | 2. years | 3. years |
| e. Are you a passive smoker at home or work? | 1. | Home: | i.  ii. | Yes  No | | |
|  | 2. | Work: | i.  ii. | Yes  No | | |

Physical Activity & Exercise

| 6. Do you exercise regularly?  *(If no, go to 7)* | 1.  2. | Yes  No | | | | |
| --- | --- | --- | --- | --- | --- | --- |
| a. What type of exercise do you participate in? (You may have more than one answer) | 1.  aerobic exercise | | 2. stretching exercise | 3. walking slowly (level ground) | 4.  Tai chi / yoga | 5.  others: _________ |
| b. How many times do you do each exercise each week? | 1.  times/wk | | 2.  times/wk | 3.  times/wk | 4.  times/wk | 5.  times/wk |
| c. What is the average duration each time? | 1._____min  a. <15 mins  b.15-30 mins  c. >30 mins | | 2._____min  a. <15 mins  b.15-30 mins  c. >30 mins | 3._____min  a. <15 mins  b.15-30 mins  c. >30 mins | 4._____min  a. <15 mins  b.15-30 mins  c. >30 mins | 5._____min  a. <15 mins  b.15-30 mins  c. >30 mins |

**Demographic data:**

| 7. What is your marital status? | 1. Single | | | 2. Married | | | | | | 3. Separated or divorced | | | 4. Widowed | | | | | 5. Refuse to answer | |
| --- | --- | --- | --- | --- | --- | --- | --- | --- | --- | --- | --- | --- | --- | --- | --- | --- | --- | --- | --- |
|  |  | | | |  | | |  | | | | | |  | | |  | | |
| 8. What is the average monthly income of your household from all sources?  $________________ | 1.  Less than $2,000 | 2.  $2,000  |  $3,999 | | | | 3.  $4,000  |  $5,999 | 4.  $6,000  |  $7,999 | | | | 5.  $8,000  |  $9,999 | | | | 6.  $10,000  |  $14,999 | 7.  $15,000  |  $19,999 | | | 8.  $20,000  |  $24,999 |
|  | 9.  $25,000  \|  $29,999 | 10.  $30,000  \|  $39,999 | | | | 11.  $40,000  \|  $59,999 | 12.  $60,000  or  above | | | | 13.  No income | | | | 14.  Refuse to  answer | 15.  Don’t know | | |  |
|  |  | | | |  | | |  | | | | | |  | | |  | | |
| 9. What is your average monthly income, including all sources?  $________________ | 1.  Less than $2,000 | | 2.  $2,001  |  $5,000 | | | | | | 3.  $5,001  |  $9,999 | | | 4.  $10,000  |  $14,999 | | | | | | 5.  $15,000  |  $19,999 | |
|  | 6.  $20,000  \|  $29,999 | | 7.  $30,000  or  above | | | | | | 8.  No income | | | 9.  Refuse to answer | | | | | | 10.  Don’t know | |

**《評估慢性疾病管理計劃成效**

**–病人自强計劃》**

**Follow up Survey**

您好！＿＿先生/小姐。我係香港大學家庭醫學及基層醫療學系嘅訪問員___________（請報自己姓名）。我地既同事大概六個月前同您做過一個**病人自强計劃**既成效既研究，依家想再請您幫忙回答一些問題。我地只會阻您大約十至十五分鐘時間。

所有個人資料係會絕對保密。（如您對是項研究有疑問，歡迎起辦公時間打電話2552-5756到香港大學家庭醫學及基層醫療學系林博士查詢。）好多謝您嘅幫忙同合作。

|  |  |
| --- | --- |
|  |  |
|  |  |
|  |  |
|  |  |
|  |  |
| **訪問日期:** |  |
|  |  |
| **訪問員編號:** |  |
|  |  |

**標準十二題簡明健康狀況調查表-第**二**版**

**說明：這項調查是詢問您對自己健康狀況的了解。此項資料記錄您的自我感覺和日常生活的情況。**

**請在一個方格內填上X號來回答每個問題。如果您不肯定怎樣回答，請按照您的理解選擇最合適的答案。**

**1. 總括來說，您認為您的健康狀況是：**

| 極好 | | 很好 | | 好 | | 一般 | | 差 |
| --- | --- | --- | --- | --- | --- | --- | --- | --- |
| 1 | 2 | | 3 | | 4 | | 5 | |

**2. 下列問題是您日常生活中可能進行的活動。以您目前的健康狀況，您在進行這些活動時，有沒有受到限制？如果有的話，程度如何？**

|  | 有很大限制 | 有一點限制 | 沒有任何限制 |
| --- | --- | --- | --- |
| a. 中等強度的活動，比如搬桌子，使用吸塵器清潔地面，玩保齡球或打太極拳 | 1 | 2 | 3 |
| b. 上幾層樓梯 | 1 | 2 | 3 |

**3. 在過去四個星期裏，您在工作或其它日常活動中，有多少時間會因為身體健康的原因而遇到下列的問題？**

|  | 常常如此 | 大部分時間 | 有時 | 偶爾 | 從來沒有 |
| --- | --- | --- | --- | --- | --- |
| a. 實際做完的比想做的要少 | 1 | 2 | 3 | 4 | 5 |
| b. 工作或其它活動的種類受到限制 | 1 | 2 | 3 | 4 | 5 |

**4. 在過去的四個星期裏，您在工作或其它日常活動中，有多少時間由於情緒方面的原因（比如感到沮喪或焦慮）遇到下列的問題？**

|  | 常常如此 | 大部分時間 | 有時 | 偶爾 | 從來沒有 |
| --- | --- | --- | --- | --- | --- |
| a. 實際做完的比想做的要少 | 1 | 2 | 3 | 4 | 5 |
| b. 工作時或從事其它活動時不如往常細心了 | 1 | 2 | 3 | 4 | 5 |

**5. 在過去四個星期裏，您身體上的疼痛對您的日常工作（包括上班和家務）有多大影響？**

| 毫無影響 | | 有很少影響 | 有一些影響 | | 有較大影響 | | 有極大影響 |
| --- | --- | --- | --- | --- | --- | --- | --- |
| 1 | 2 | | 3 | 4 | | 5 | |

**6. 下列問題是有關您在過去四個星期裏您覺得怎樣和您其它的情況。針對每一個問題，請選擇一個最接近您的感覺的答案。在過去四個星期裏有多少時間：**

|  | 常常如此 | 大部分時間 | 有時 | 偶爾 | 從來沒有 |
| --- | --- | --- | --- | --- | --- |
| a. 您感到心平氣和？ | 1 | 2 | 3 | 4 | 5 |
| b. 您感到精力充足？ | 1 | 2 | 3 | 4 | 5 |
| c. 您覺得心情不好，悶悶不樂？ | 1 | 2 | 3 | 4 | 5 |

**7. 在過去四個星期裏，有多少時間由於您的身體健康或情緒問題妨礙了您的社交活動 (比如探親、訪友等）？**

| 常常有妨礙 | | 大部分時間有妨礙 | | 有時有妨礙 | | 偶爾有妨礙 | | 完全沒有妨礙 |
| --- | --- | --- | --- | --- | --- | --- | --- | --- |
| 1 | 2 | | 3 | | 4 | | 5 | |

**病人自强量表**

參加了共同護理計劃後,您感到以下的情況有何改變?

(請於每行出最適合的答案)

|  | **大為改善** | **有所改善** | **一樣** | **少了** | **不適用** |
| --- | --- | --- | --- | --- | --- |
|  | **2** | **1** | **0** | **0** |  |
| a. 能夠應付生活 |  |  |  |  |  |
|  |  |  |  |  |  |
| b. 能夠了解自己的疾病 |  |  |  |  |  |
|  |  |  |  |  |  |
| c. 能夠面對自己的疾病 |  |  |  |  |  |
|  |  |  |  |  |  |
| d. 能夠保持自己身體健康 |  |  |  |  |  |
|  | **大為增强** | **有所增强** | **無甚改變** | **少了** | **不適用** |
| e. 對自己健康的信心 |  |  |  |  |  |
|  |  |  |  |  |  |
| f. 擁有自助的能力 |  |  |  |  |  |

**整體健康轉變評估問卷**

參加了共同護理計劃後, 您認為您的身體健康情況比之前是 ……

(請出最適合的答案)

| 好了很多 | 好了 | 好了一點 | 一樣 | 差了一點 | 差了 | 差了很多 |
| --- | --- | --- | --- | --- | --- | --- |
| +3 | +2 | +1 | 0 | -1 | -2 | -3 |

多謝您參與研究！

-------------------------------------------------完-----------------------------------------------

**私家醫療服務的使用**

| 1. **在過去四個星期裏，**你有冇睇過私家醫生 (西醫 / 中醫)？  *(如答沒有, 回答問題2)* | 1.  2. | | | 有  沒有 | | | | | | | | | |
| --- | --- | --- | --- | --- | --- | --- | --- | --- | --- | --- | --- | --- | --- |
|  | 1. | 普通科 | | | | 2. | 專科 | | | 3. | 中醫 | | |
| a. 請問你睇過私家醫生幾多次？ | 1. | | | | 次 | 2. | 次 | | | 3. | 次 | | |
| b. 請問你由屋企**平均**要搭幾耐車先到私家診所呢？ | 1. | | | | 分鐘/小時 | 2. | 分鐘/小時 | | | 3. | 分鐘/小時 | | |
| c. 請問你由屋企搭車去私家診所要幾多錢呢？ | 1. | | | |  | 2. |  | | | 3. |  | | |
| d. 請問你睇一次私家醫生**平均**要幾耐？ (包括等候同埋見醫生嘅時間) | 1. | | | | 分鐘/小時 | 2. | 分鐘/小時 | | | 3. | 分鐘/小時 | | |
| e. **每次**求診嘅費用有幾多  (包括僱主或保險公司嘅補貼)？ | 1. | | | |  | 2. |  | | | 3. |  | | |
| f. 有冇人陪你睇私家醫生呢？佢係邊個？  (性別：男/女) | 1. | | i.  ii. | | 有 ( 男/女 )  沒有 | 2. | | i.  ii. | 有 ( 男/女 )  沒有 | 3. | | i.  ii. | 有 ( 男/女 )  沒有 |
| g. 佢有冇請假呢？ | 1. | | i.  ii. | | 有  沒有 | 2. | | i.  ii. | 有  沒有 | 3. | | i.  ii. | 有  沒有 |

| 2. **在過去四個星期裏，**你有冇試過自己買藥食？  *(如答沒有, 回答問題3)* | 1.  2. | 有  沒有 | |
| --- | --- | --- | --- |
| 怎樣處理，幾多次，**每次**幾多錢？ | 次數 | | 每次費用 ($) |
| 1. 西藥成藥 | 1. | | 2. |
| b. 中藥成藥 (例如：牛黃解毒片、銀翹) | 1. | | 2. |
| c. 中草藥 (藥材舖執藥) | 1. | | 2. |
| 1. 其他，請註明：______________ | 1. | | 2. |

| 3. **在過去六個月裏，**你有冇入住私家醫院？  *(如答沒有, 回答問題4)* | 1.  2. | 有  沒有 |
| --- | --- | --- |
| a. 請問你留左幾多日院呢？ | 日 | |
| b. 請問你由屋企**平均**要搭幾耐車先到私家醫院呢？ | 分鐘/小時 | |
| c. 請問你由屋企搭車去私家醫院要幾多錢呢？ | $ | |
| d. **上一次**入住醫院嘅費用有幾多  (包括僱主或保險公司嘅補貼)？ | $ | |
| e. 有冇人係你**上一次**入院時照顧患病嘅你？佢係邊個？ (性別：男/女) | 1.  2. | 有 _____ (男/女)  沒有 |
| f. 係你**上一次**入院時佢請咗幾多日假照顧你？ | 日 | |

**家居照顧**

| 4. **在過去六個月裏，**當你患病嘅時候有冇人照顧你嘅起居飲食？佢係邊個？ (性別：男/女) | 1.  2. | 有 _____ (男/女)  沒有 | | |
| --- | --- | --- | --- | --- |
| 4a. 佢洗唔洗請假照顧你？唔番工定係唔番學？如果要請假，請幾多日呢？ | 1. | 有 | i.  ii. | 唔番工________日  唔番學 ________日 |
|  | 2. | 沒有 | | |

**生活方式**

吸煙

| 5. 你有冇食煙嘅習慣？*(如答沒有, 回答問題****5e****)* | 1.  2. | | 有  沒有 | | | | |
| --- | --- | --- | --- | --- | --- | --- | --- |
| a. 請問你食煙嘅習慣是： | 1. 現在完全戒煙 | | | | | 2. 現在間中食煙，每日不夠一支 | 3. 現在每日食煙起碼一支 |
| b. 請問你已戒煙有幾耐？ | ___年___ 月 | | | | |  |  |
| c. 請問你(以前 / 現在)平均每日吸多少支煙? | 1. 支 | | | | | 2. 支 | 3. 支 |
| d. 請問你食左幾多年煙？ | 1. 年 | | | | | 2. 年 | 3. 年 |
| e. 不計算你自己，請問你**屋企**同埋**工作範圍**有沒有其他人食煙呢？ | 1. | 屋企: | | i.  ii. | 有  沒有 | | |
|  | 2. | 工作範圍: | | i.  ii. | 有  沒有 | | |

運動習慣

| 6. 你有冇定期做運動嘅習慣？  *(如答沒有, 回答問題7)* | 1.  2. | 有  沒有 | | | | |
| --- | --- | --- | --- | --- | --- | --- |
| a. 你是參與什麼類型的體育活動(你可選擇多過一個答案) | 1.有氧健身法 | | 2. 舒展運動 | 3. 平路慢步 | 4. 太極/瑜珈 | 5. 其他： _________ |
| b. 每星期做幾多次運動？ | 1. ____ 次 | | 2. _____ 次 | 3._____ 次 | 4._____ 次 | 5. _____ 次 |
| c. 你每次平均做幾耐運動？ | 1._____分鐘  a. < 15 分鐘  b. 15-30分鐘  c. > 30分鐘 | | 2._____分鐘  a. < 15 分鐘  b. 15-30分鐘  c. > 30分鐘 | 3._____分鐘  a. < 15 分鐘  b. 15-30分鐘  c. > 30分鐘 | 4._____分鐘  a. < 15 分鐘  b. 15-30分鐘  c. > 30分鐘 | 5.____分鐘  a. < 15 分鐘  b. 15-30分鐘  c. > 30分鐘 |

----------------------------------------------------完-------------------------------------------------

**SF-12 HEALTH SURVEY (VERSION 2.0)**

INSTRUCTIONS: This survey asks for your views about your health. This information will help keep track of how you feel and how well you are able to do your usual activities.

Answer every question by marking the answer as indicated. If you are unsure about how to answer a question, please give the best answer you can.

1. In general, would you say your health is:

| Excellent | Very good | Good | Fair | Poor |
| --- | --- | --- | --- | --- |
| 1 | 2 | 3 | 4 | 5 |

2. The following questions are about activities you might do during a typical day. Does **your health now limit you** in these activities? If so, how much?

|  | Yes, limited a lot | Yes, limited a little | No, not limited at all |
| --- | --- | --- | --- |
| a. **Moderate activities**, such as moving a table, pushing a vacuum cleaner, bowling, or playing golf | 1 | 2 | 3 |
| b. Climbing **several** flights of stairs | 1 | 2 | 3 |

3. During the **past 4 weeks**, how much of the time have you had any of the following problems with your work or other regular daily activities **as a result of your physical health**?

|  | All of the time | Most of the time | Some of the time | A little of the time | None of the time |
| --- | --- | --- | --- | --- | --- |
| a. **Accomplished less** than you would like | 1 | 2 | 3 | 4 | 5 |
| b. Were limited in the **kind** of work or other activities | 1 | 2 | 3 | 4 | 5 |

4. During the **past 4 weeks**, how much of the time have you had any of the following problems with your work or other regular daily activities **as a result of any emotional problems** (such as feeling depressed or anxious)?

|  | All of the time | Most of the time | Some of the time | A little of the time | None of the time |
| --- | --- | --- | --- | --- | --- |
| a. **Accomplished less** than you would like | 1 | 2 | 3 | 4 | 5 |
| b. Did work or other activities less **carefully** than usual | 1 | 2 | 3 | 4 | 5 |

5. During the **past 4 weeks**, how much did **pain** interfere with your normal work (including both work outside the home and housework)?

| Not at all | A little bit | Moderately | Quite a bit | Extremely |
| --- | --- | --- | --- | --- |
| 1 | 2 | 3 | 4 | 5 |

6. These questions are about how you feel and how things have been with you during the **past 4 weeks**. For each question, please give the one answer that comes closest to the way you have been feeling. How much of the time during the **past 4 weeks**...

|  | All of the time | Most of the time | Some of the time | A little of the time | None of the time |
| --- | --- | --- | --- | --- | --- |
| a. Have you felt calm and peaceful? | 1 | 2 | 3 | 4 | 5 |
| b. Did you have a lot of energy? | 1 | 2 | 3 | 4 | 5 |
| c. Have you felt downhearted and depressed? | 1 | 2 | 3 | 4 | 5 |

7.During the **past 4 weeks**, how much of the time has your **physical health** or **emotional problems** interfered with your social activities (like visiting friends, relatives, etc.)?

| All of the time | Most of the time | Some of the time | A little of the time | None of the time |
| --- | --- | --- | --- | --- |
| 1 | 2 | 3 | 4 | 5 |

**The Patient Enablement Instrument**

As a result of your participation in Shared-Care Programme, do you feel you are….

(please 🗸 one box in each row):-

**MUCH NOT**

**BETTER BETTER SAME LESS APPLICABLE**

**2 1 0 0**

able to cope with life

able to understand your illness

able to cope with your illness

able to keep yourself healthy

**MUCH NOT**

**MORE MORE SAME LESS APPLICABLE**

**2 1 0 0**

confident about your health

able to help yourself

**Global Rating on Change Scale**

How would you rate the change in your health condition after participating in the Shared Care Programme?

| **MUCH BETTER** | **BETTER** | **A LITTLE BETTER** | **SAME** | **A LITTLE WORSE** | **WORSE** | **MUCH**  **WORSE** |
| --- | --- | --- | --- | --- | --- | --- |
| +3 | +2 | +1 | 0 | -1 | -2 | -3 |

-------------------------------------------------end-----------------------------------------------

**Utilisation in private medical services:**

| 1. Did you have any private doctor consultation in the **last 4 weeks (western medicine / Chinese medicine)**? *(If no, go to 2)* | 1.  2. | Yes  No | | | | | | | |
| --- | --- | --- | --- | --- | --- | --- | --- | --- | --- |
|  |  | General  Practitioner | |  | Specialist | |  | Chinese Medicine | |
| a. How many visits? | 1. | visits | | 2. | visits | | 3. | visits | |
| b. How long did you spend for travelling from home to clinic **on average**? | 1. | Min/Hrs | | 2. | Min/Hrs | | 3. | Min/Hrs | |
| c. How much did you spend for travelling from home to clinic? | 1. |  | | 2. |  | | 3. |  | |
| d. How long did you stay in the clinic **on average**? (Waiting time and consultation time) | 1. | Min/Hrs | | 2. | Min/Hrs | | 3. | Min/Hrs | |
| e. How much did you pay for **each visit** (including subsidies from employer or insurance company)? | 1. |  | | 2. |  | | 3. |  | |
| f. Did any person accompany with you to go for visit(s)? Who was that person? (Gender: Male /Female) | 1. | i.  ii. | Yes (M/F) No | 2. | i.  ii. | Yes(M/F)  No | 3. | i.  ii. | Yes (M/F) No |
| g. Did she/he take time off work/ study? | 1. | i.  ii. | Yes (M/F)  No | 2. | i.  ii. | Yes (M/F)  No | 3. | i.  ii. | Yes (M/F)  No |

| 2. Did you get any self medication in the **last 4 weeks**?  *(If no, go to 3)* | 1.  2. | Yes  No | |
| --- | --- | --- | --- |
| How many times did you get self medication and how much did you pay **each time**? | No. of times | | Cost each time ($) |
| 1. Western over-the-counter medicine | 1. | | 2. |
| b. Chinese over-the-counter medicine  (eg. Chinese medical products) | 1. | | 2. |
| c. Chinese herbal medicine  (eg. herbal medicines which require boiling) | 1. | | 2. |
| 1. Others, please specify:_________________ | 1. | | 2. |

| 3. Did you have any private hospitalisation in the **last 6 months**?  *(If no, go to 4)* | 1.  2. | Yes  No |
| --- | --- | --- |
| a How many days of admission? | days | |
| b. How long did you spend for travelling from home to hospital **on average**? | Min/Hrs | |
| c. How much did you spend for travel from home to hospital? | $ | |
| d. How much did you pay for the **last admission** (including subsidies from employer or insurance company)? | $ | |
| e. Did any person visit you during your **last admission**? Who was that person?  (Gender: Male /Female) | 1.  2. | Yes ( M / F )  No |
| f. How many days did she/he take time off work because of visiting you during your **last admission**? | days | |

**Home care cost:**

| 4. Did any person take care of you at home while you’re sick in the last 6 months? If so, who was that person? (Gender: Male/Female) | 1.  2. | Yes ( M / F )  No | | |
| --- | --- | --- | --- | --- |
| 4a. Did she/ he take any time off work/ study to take care of you? If so, how many days? | 1. | Yes | i.  ii. | Work ________days  study ________days |
|  | 2. | No | | |

**Lifestyle**:

Smoking

| 5. Did/do you have a habit of smoking? *(If no, go to* ***5e****)* | 1.  2. | Yes  No | | | | |
| --- | --- | --- | --- | --- | --- | --- |
| a. Your smoking habit is: | 1. Now have quit already  (former smoker) | | | | 2. Currently smoke occasionally, fewer than one cigarette a day | 3. Currently smoke, at least one cigarette a day |
| b. How long have you quitted smoking? | _____Yr(s) ____ Mth(s) | | | |  |  |
| c. How many cigarettes (past / present) on average did/do you smoke a day? | 1. cigarettes | | | | 2. cigarettes | 3. cigarettes |
| d. How many years did you/have you smoked? | 1. years | | | | 2. years | 3. years |
| e. Are you a passive smoker at home or work? | 1. | Home: | i.  ii. | Yes  No | | |
|  | 2. | Work: | i.  ii. | Yes  No | | |

Physical Activity & Exercise

| 6. Do you exercise regularly?  *(If no, go to 7)* | 1.  2. | Yes  No | | | | |
| --- | --- | --- | --- | --- | --- | --- |
| a. What type of exercise do you participate in? (You may have more than one answer) | 1.  aerobic exercise | | 2. stretching exercise | 3. walking slowly (level ground) | 4.  Tai chi / yoga | 5.  others: _________ |
| b. How many times do you do each exercise each week? | 1.  times/wk | | 2.  times/wk | 3.  times/wk | 4.  times/wk | 5.  times/wk |
| c. What is the average duration each time? | 1._____min  a. <15 mins  b.15-30 mins  c. >30 mins | | 2._____min  a. <15 mins  b.15-30 mins  c. >30 mins | 3._____min  a. <15 mins  b.15-30 mins  c. >30 mins | 4._____min  a. <15 mins  b.15-30 mins  c. >30 mins | 5._____min  a. <15 mins  b.15-30 mins  c. >30 mins |

-------------------------------------------------end---------------------------------------------
